# Mapping QTL for white striping in relation to breast muscle yield and meat quality traits in broiler chickens

**DOI:** 10.1186/s12864-018-4598-9

**Published:** 2018-03-20

**Authors:** Eva Pampouille, Cécile Berri, Simon Boitard, Christelle Hennequet-Antier, Stéphane A. Beauclercq, Estelle Godet, Christophe Praud, Yves Jégo, Elisabeth Le Bihan-Duval

**Affiliations:** 1 BOA, INRA, Université de Tours, 37380 Nouzilly, France; 2 Hubbard SAS, 22800 Mauguérand, Le Foeil-Quintin France; 3 GenPhySE, Université de Toulouse, INRA, INPT, INP-ENVT, 31320 Castanet Tolosan, France

**Keywords:** White striping, Meat quality, QTL, eQTL, GWAS, Chicken

## Abstract

**Background:**

White striping (WS) is an emerging muscular defect occurring on breast and thigh muscles of broiler chickens. It is characterized by the presence of white striations parallel to the muscle fibers and has significant consequences for meat quality. The etiology of WS remains poorly understood, even if previous studies demonstrated that the defect prevalence is related to broiler growth and muscle development. Moreover, recent studies showed moderate to high heritability values of WS, which emphasized the role of genetics in the expression of the muscle defect. The aim of this study was to identify the first quantitative trait loci (QTLs) for WS as well as breast muscle yield (BMY) and meat quality traits using a genome-wide association study (GWAS). We took advantage of two divergent lines of chickens selected for meat quality through *Pectoralis major* ultimate pH (pHu) and which exhibit the muscular defect. An expression QTL (eQTL) detection was further performed for some candidate genes, either suggested by GWAS analysis or based on their biological function.

**Results:**

Forty-two single nucleotide polymorphisms (SNPs) associated with WS and other meat quality traits were identified. They defined 18 QTL regions located on 13 chromosomes. These results supported a polygenic inheritance of the studied traits and highlighted a few pleiotropic regions. A set of 16 positional and/or functional candidate genes was designed for further eQTL detection. A total of 132 SNPs were associated with molecular phenotypes and defined 21 eQTL regions located on 16 chromosomes. Interestingly, several co-localizations between QTL and eQTL regions were observed which could suggest causative genes and gene networks involved in the variability of meat quality traits and BMY.

**Conclusions:**

The QTL mapping carried out in the current study for WS did not support the existence of a major gene, but rather suggested a polygenic inheritance of the defect and of other studied meat quality traits. We identified several candidate genes involved in muscle metabolism and structure and in muscular dystrophies. The eQTL analyses showed that they were part of molecular networks associated with WS and meat quality phenotypes and suggested a few putative causative genes.

**Electronic supplementary material:**

The online version of this article (10.1186/s12864-018-4598-9) contains supplementary material, which is available to authorized users.

## Background

During the past few decades, a notable increase in the demand for poultry meat has been observed due to its convenience for cooking and processing, health benefits, and low price. To meet market demand and the world population increase, producers have had to increase their production while reducing the costs. Consequently, the production of broiler chickens has become more efficient mainly thanks to genetic selection. By comparing two genetic strains representative of the broilers being grown in 1957 or in 2001, Havenstein et al. [1] showed that changes over these 44 years resulted in a 2001 broiler that required approximately one-third the time and over a threefold decrease in the amount of feed consumed to reach the market weight of 1,8 kg. Genetic selection brought about 85% to 90% of the change that has occurred in broiler growth rate [1]. Genetics was also the major contributor to changes in the yield of carcass parts that continued to increase over time especially for breast meat yield which currently exceeds one fifth of the weight of the bird [2, 3]. Nevertheless, for nearly a decade now, the poultry industry in many countries (i.e. Italy, the United States, Brazil, the United Kingdom, Finland, France, etc.) has witnessed an increasing prevalence of broiler breast muscle abnormalities such as WS [4].

WS is characterized by white striations parallel to muscle fibers, mainly on breast, but sometimes on thigh and tender muscle of broilers. Histological observations of white-striped meat indicate an increase in degenerative fibers associated with regeneration phenomenon (nuclear rowing, multiple internationalized nuclei), variation in muscle fiber size, mononuclear cell infiltration, adiposis, and fibrosis [5]. This myopathic pattern has significant consequences for meat quality. WS fillets have a higher fat content and a lower protein content than normal fillets, resulting in meat of reduced nutritional quality [6–10]. They also exhibit higher cooking loss (CL), as a result of a lower water holding capacity (WHC), and reduced tenderness [10–13]. In addition to altering the sensory and technological properties of meat, the severity of WS has negative effects on breast fillet appearance and therefore on consumer purchases [14]. Furthermore, meat affected by breast muscle myopathies can be downgraded or in some cases condemned, leading to serious economic losses. This muscular defect could also result in social acceptability issues regarding animal welfare and meat quality [15].

WS etiology remains poorly understood even if previous studies demonstrated that the prevalence of this defect is directly related to broiler growth performances [6, 11, 16, 17]. Bailey et al. provided the first estimates of genetic parameters of chicken breast muscle myopathies (WS, wooden breast, deep pectoral myopathy) recorded in a high-yielding line or a moderate-yielding line [18]. WS was the most common myopathy in both chicken lines. It was also the most heritable one, with heritability values ranging from 0.19 to 0.34. Although WS incidence was significantly higher in the high-yielding line than in the moderate-yielding line (49,6% vs 14,5%), the estimated genetic correlations with body weight (BW) and breast muscle yield (BMY) remained low to moderate (0.06 to 0.23) in the two studied lines [18]. The second study conducted by Alnahhas et al. showed a strong genetic basis of WS (h^2^ = 0.65) measured in two divergent lines of broilers selected for meat quality through *Pectoralis major* pHu [19]. A high genetic correlation between WS and BMY was reported (rg = 0.68) while it was moderate with BW [19]. Difference in magnitude of genetic parameters exist between the two studies (related to differences of genetic backgrounds or of methods of estimation), but they both indicate a role of genetic factors in the expression of WS. However, no information is currently available regarding the genomic architecture of this trait.

The aim of this GWAS was to identify QTL for WS as well as BMY and meat quality traits by taking advantage of the two divergent lines of broilers selected for *Pectoralis major* pHu and which exhibit the muscular defect. A previous study showed that there is a higher incidence of WS in line with high pHu value [19]. One hypothesis is that the depletion of glycogen reserves observed in the high pHu line would be a predisposing metabolic environment for WS development.

After the first step of QTL mapping, an eQTL detection was performed for a few positional and/or functional candidate genes. This made it possible to better understand the associated molecular mechanisms, and provided candidate genes and markers that could be used in breeding programs to reduce the prevalence of WS and, more generally, to improve meat quality in broilers.

## Results and discussion

### Descriptive statistics

The descriptive statistics describing the distribution of carcass composition and meat quality traits of the 558 broilers used for the current GWAS are shown in Table 1. A logarithmic transformation was applied to the thiobarbituric acid-reactive substance index (TBA-RS), a marker of lipid peroxidation, in order to normalize its distribution. A chi^2^ homogeneity test was performed to characterize the distribution of normal fillets, fillets that were moderately affected, and fillets severely affected by WS defect between lines (pHu + and pHu-). The proportion of fillets with each degree of WS severity was not the same in both lines (*p* = 2.6 × 10^− 15^). There was a higher proportion of normal fillets in the pHu- line than in the pHu + line (57.3% vs 32.4%, respectively) and incidence of severe WS was higher in the pHu + line than in the pHu- line (27.7% vs 4.3%, respectively) (*p* = 7.5 × 10^− 16^).

**Table 1:** Means and standard deviation for body composition and meat quality traits measured at 6 weeks

Traits*	Number	pHu +		pHu -	
		Male	Female	Male	Female
		(*N* = 135)	(*N* = 143)	(*N* = 118)	(*N* = 162)
BW (g)	558	2990 ± 298	2588 ± 222	2995 ± 327	2631 ± 186
PMY (%)	558	8.45 ± 0.85	8.73 ± 0.67	8.28 ± 0.64	8.36 ± 0.55
PmY (%)	555	1.86 ± 0.15	2.00 ± 0.16	1.83 ± 0.13	1.94 ± 0.12
BMY (%)	555	20.63 ± 1.84	21.46 ± 1.43	20.21 ± 1.43	20.61 ± 1.24
TY (%)	556	23.08 ± 1.31	22.40 ± 1.13	22.67 ± 1.13	22.10 ± 0.97
AFP (%)	556	1.73 ± 0.34	2.06 ± 0.38	1.70 ± 0.31	2.03 ± 0.34
L*	556	44.98 ± 3.90	44.75 ± 3.41	53.09 ± 2.91	52.65 ± 3.10
a*	556	−0.28 ± 0.61	−0.19 ± 0.64	0.03 ± 0.63	0.06 ± 0.49
b*	554	10.79 ± 1.37	10.72 ± 1.28	12.84 ± 1.20	12.70 ± 1.10
DL (%)	558	2.14 ± 1.52	1.91 ± 1.08	4.42 ± 1.49	4.19 ± 1.40
CL (%)	555	9.40 ± 2.40	8.80 ± 1.81	11.19 ± 2.18	10.34 ± 1.76
CCY (%)	536	85.81 ± 3.86	86.73 ± 3.36	83.26 ± 4.10	83.72 ± 4.10
SF (N/cm^2^)	556	11.44 ± 2.14	10.85 ± 2.08	16.44 ± 2.78	15.77 ± 2.77
IFP (%)	555	1.54 ± 0.47	1.37 ± 0.37	1.58 ± 0.39	1.33 ± 0.35
TBA-RS	554	0.35 ± 0.42	0.28 ± 0.34	0.57 ± 0.42	0.45 ± 0.37
WS (%)	557	61.5	73.4	42.7	42.6

^a^*BW* body weight*, PMY Pectoralis major* yield, *PmY Pectoralis minor* yield, *BMY* breast meat yield, *TY* thigh yield, *AFP* abdominal fat percentage, L* lightness, a* redness, b* yellowness, *DL* drip loss*, CL* cooking losses, *CCY* curing-cooking yield, *SF* shear force, *IFP* intramuscular fat content, *TBA-RS* thiobarbituric acid-reactive substance, *WS* white striping

These results are in agreement with previous studies reporting that meat with myopathy is often characterized by a higher pHu value than normal meat [11, 12, 19–22].

### QTL detection

#### White striping

**Fig. 1:**
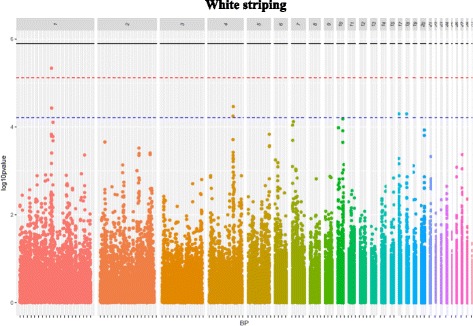
Manhattan plot showing the association of SNPs with WS. Black line represents the 5% genome-wide threshold, red line the 5% GGA1-wide threshold, blue line the 5% GGA17 and GGA18-wide threshold

The GWAS performed on the whole population created from the two divergent lines identified three SNPs that were significantly associated with WS at the chromosome level and located on GGA1, GGA17, and GGA18 (Fig. 1, Table 2). This finding was not in favor of a monogenic inheritance of WS since several QTLs seemed to be involved in addition to the polygenic effect included in the model (see Methods section). Moreover, no co-localization with SNPs previously identified for pHu in the same genetic population was observed [23], which is not in favor of common genetic variants between the two traits. In order to explore whether pHu could indirectly influence WS, intra-line QTL detection of WS was performed (Additional file 1: Figure S1). No significant SNPs were detected in the pHu- line, while eight SNPs were significant at the chromosome threshold in the pHu + line (Table 3). They confirmed the two regions previously detected on GGA1 and GGA17 in the entire population and identified a new one on GGA20. The SNP identified with the lowest *p*-value was also the most significant SNP associated with WS in the entire population (Gga_rs13899127 on GGA1). Five SNPs significant at the GGA1 threshold co-localized with this SNP. Furthermore, we recovered the same SNP on GGA17 as the one associated with WS in the entire population (Gga_rs15804842).

**Table 2:** Significant SNPs for white striping, body composition, and meat quality traits

Traits^a^	QTL^b^	GGA	SNP ID	Position^c^	P-value^d^
L*	QTL1	1	Gga_rs13841646	26,417,615	7.27 × 10^− 6^
L*	QTL1	1	GGaluGA009377	26,433,696	7.27 × 10^− 6^
L*	QTL1	1	Gga_rs15215403	26,454,554	7.27 × 10^− 6^
WS	QTL2	1	Gga_rs13899127	87,535,862	4.62 × 10^− 6^
PMY	QTL3	4	GGaluGA263381	65,974,471	6.96 × 10^−6^
BMY	QTL3	4	GGaluGA263381	65,974,471	9.13 × 10^−6^
CL	QTL4	4	Gga_rs16454334	90,828,977	8.38 × 10^−6^
CL	QTL4	4	GGaluGA271193	90,877,143	1.39 × 10^−6^
CL	QTL4	4	GGaluGA271212	90,940,405	7.30 × 10^−6^
CL	QTL4	4	GGaluGA271252	91,016,447	1.12 × 10^− 5^
CL	QTL4	4	Gga_rs13776465	91,056,759	6.05 × 10^−6^
CL	QTL4	4	Gga_rs16454745	91,098,157	**1.02** × **10**^**−8**^
DL	QTL4	4	Gga_rs16454745	91,098,157	5.15 × 10^−6^
L*	QTL4	4	Gga_rs16454745	91,098,157	9.01 × 10^−6^
b*	QTL4	4	Gga_rs16454745	91,098,157	1.59 × 10^− 5^
CL	QTL4	4	Gga_rs14506759	91,113,465	**2.27** × **10**^**−7**^
DL	QTL4	4	Gga_rs14506759	91,113,465	2.32 × 10^−6^
L*	QTL4	4	Gga_rs14506759	91,113,465	5.51 × 10^−6^
b*	QTL4	4	Gga_rs14506759	91,113,465	1.17 × 10^− 5^
CL	QTL4	4	Gga_rs15648179	91,195,923	**1.76** × **10**^**− 9**^
DL	QTL4	4	Gga_rs15648179	91,195,923	1.70 × 10^−6^
PMY	QTL5	5	Gga_rs14515278	11,832,057	1.99 × 10^− 5^
DL	QTL6	5	Gga_rs13794130	20,549,458	1.64 × 10^−6^
DL	QTL6	5	GGaluGA278338	20,902,598	1.81 × 10^− 5^
CL	QTL6	5	Gga_rs16475257	20,970,166	7.14 × 10^−6^
PmY	QTL7	5	GGaluGA289933	53,765,237	1.23 × 10^− 5^
SF	QTL8	5	Gga_rs14554568	56,426,990	2.26 × 10^− 5^
PMY	QTL9	8	GGaluGA333125	28,864,644	1.79 × 10^− 5^
a*	QTL10	12	Gga_rs14032735	3,153,630	3.59 × 10^− 5^
a*	QTL10	12	Gga_rs14033649	3,956,551	3.67 × 10^− 5^
b*	QTL11	14	GGaluGA101062	5,003,931	5.36 × 10^− 5^
WS	QTL12	17	Gga_rs15804842	2,376,482	5.07 × 10^− 5^
WS	QTL13	18	Gga_rs14108368	2,766,387	5.02 × 10^− 5^
PmY	QTL14	19	Gga_rs14115815	1,020,966	5.39 × 10^− 5^
PmY	QTL14	19	Gga_rs15044017	1,037,551	5.64 × 10^− 5^
DL	QTL15	24	Gga_rs16197733	4,335,811	1.58 × 10^− 5^
b*	QTL15	24	Gga_rs16197733	4,335,811	6.12 × 10^− 5^
a*	QTL16	25	Gga_rs15561568	1,745,235	1.14 × 10^− 4^
a*	QTL16	25	GGaluGA194453	1,793,966	6.68 × 10^− 5^
a*	QTL16	25	Gga_rs16741571	2,129,327	4.12 × 10^−5^
CCY	QTL17	26	GGaluGA197797	4,554,077	5.24 × 10^−5^
b*	QTL18	27	Gga_rs16205408	2,040,050	1.08 × 10^−4^

^a^L* lightness, *WS* white striping, *PMY Pectoralis major* yield, *BMY* breast meat yield, *CL* cooking losses, *DL* drip loss, b* yellowness, *PmY Pectoralis minor* yield, *SF* shear force, a* redness, *CCY* curing-cooking yield

^b^ Name of the QTL region

^c^ Positions are indicated on galgal5 assembly

^d^ Genome-wide significant *P*-values are indicated in bold

**Table 3:** Significant SNPs for white striping in the pHu + line

GGA	SNP ID	Position^a^	*P*-value
1	Gga_rs13899127	87,535,862	1.33 × 10^− 6^
1	Gga_rs13899410	87,752,766	2.20 × 10^− 6^
1	GGaluGA030724	87,760,003	2.20 × 10^− 6^
1	Gga_rs14856166	88,384,404	2.98 × 10^− 6^
1	Gga_rs13900230	88,416,292	2.98 × 10^− 6^
1	Gga_rs13900307	88,438,949	2.98 × 10^− 6^
17	Gga_rs15804842	2,376,482	1.11 × 10^− 5^
20	Gga_rs15177428	10,347,742	2.78 × 10^− 5^

^a^ Positions are indicated on galgal5 assembly

For this SNP, the minor allele frequency (MAF) was similar in both lines (MAF = 0.446 and MAF = 0.478 in the pHu + and pHu- lines, respectively), but the SNP effect was higher in the pHu + line than in the pHu- line (β = − 3.2 × 10^− 1^ and β = − 6.5 × 10^− 2^, respectively). This illustrated that unless no co-localization was observed between WS and pHu QTLs, there might be an interaction between the genetic control of WS and the metabolic and physiological status of the two lines.

#### Body composition and meat quality traits

Three SNPs were significant at the genome threshold, while 39 SNPs were significant at the chromosome level for body composition and meat quality traits (Table 2). These SNPs were mostly associated with CL and drip loss (DL), color parameters, and *Pectoralis major* and *minor* yields (PMY and PmY, respectively). They defined 15 QTL regions distributed over 11 chromosomes. As for WS, these results strongly suggested a polygenic inheritance of the studied carcass and meat quality parameters.

**Fig. 2:**
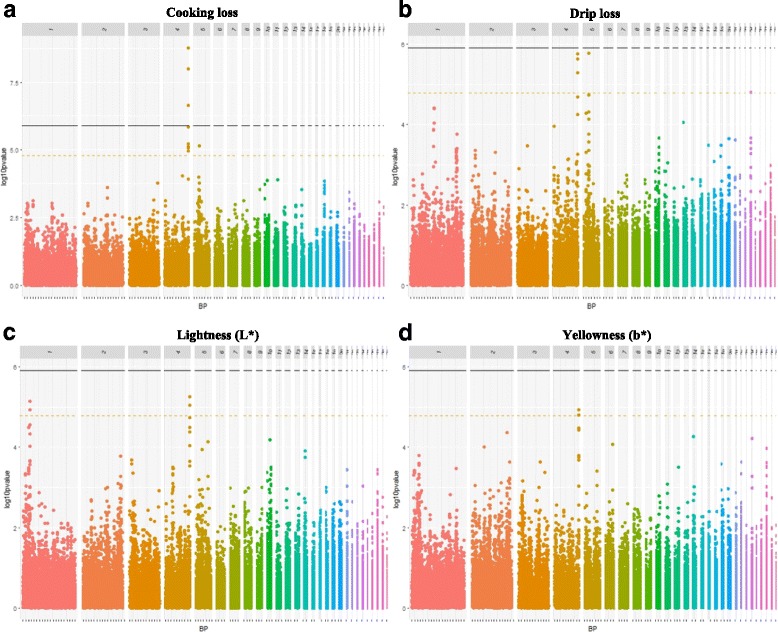
Manhattan plots showing the pleiotropic region on GGA4. Manhattan plots of CL (**a**), DL (**b**), L* (**c**) and b* (**d**). Black line represents the 5% genome-wide threshold and orange line the 5% GGA4-wide threshold

More than one third of the detected SNPs were located on GGA4 and defined two QTL regions (QTL3, QTL4). The first one (QTL3) was defined by a unique SNP (GGaluGA263381 at 65.97 Mb) which controlled both PMY and BMY. Interestingly, this SNP was also close to significance for WS (*p* = 3.5 × 10^− 5^, not shown in the table). The second one (QTL4) (90.82 to 91.20 Mb) contained three genome-wide significant SNPs for CL as well as several chromosome-wide significant SNPs for DL, lightness (L*) and yellowness (b*) of the meat. There were two common SNPs between DL, CL, L* and b* (Gga_rs1654745 at 91.09 Mb and Gga_rs14506759 at 91.11 Mb) and one more common SNP between CL and DL at 91.20 Mb (Gga_rs15648179). As illustrated in Fig. 2, the QTL4 region appeared to be a pleiotropic region controlling both the WHC (through DL and CL), and the meat color (through L* and b*). Although strong genetic correlations have already been reported between these parameters and the meat pHu in chickens [24, 25], no QTL for pHu was found nearby [23]. Similarly, we did not observe any co-localization with QTL of WS, which can also impair WHC and meat color [11].

Two additional pleiotropic regions were evidenced (QTL6, QTL15), one for DL and CL on GGA5 (20.55 to 20.97 Mb), and the other, for DL and b* on GGA24 (4.33 Mb).

A significant positive genetic relationship between WS and intramuscular fat content (IMF) had previously been reported in this population [19], suggesting that IMF could be a useful quantitative predictor of WS. However, no significant SNPs were detected for IMF in the current study, maybe because this trait was controlled by many genes with too small effects. In the same way, no QTLs were found for the measurements of TBA-RS index, a marker of lipid peroxidation.

### Identification of candidate genes

#### WS phenotype

Identification of genomic regions involved in the control of WS is an opportunity to improve our understanding of the molecular mechanisms underlying the appearance of this defect. For that, we looked for candidate genes within several regions of interest. They comprised the three significant WS-QTL regions (on GGA1, GGA17, and GGA18), the GGA4 region for which a pleiotropic effect with BMY was suggested, and two additional suggestive regions for which SNPs (Gga_rs16576992 at 4.51 Mb on GGA7 and GGaluGA071697 at 16.43 Mb on GGA10) close to significance were detected (*p* = 7.52 × 10^− 5^ and 6.60 × 10^− 5^ respectively).

Using an R software package (Gviz) [26], we visualized the gene environment around these different regions of interest (Additional file 2: Figure S2, Additional file 3: Figure S3, Additional file 4: Figure S4, Additional file 5: Figure S5 and Additional file 6: Figure S6). Potential candidate genes were selected based on their proximity with a significant SNP and/or their biological function (Table 4). Most of the selected genes are involved in muscular structure and processes related to muscle fiber regeneration and repair (i.e., *MYH15, MYH1E*, *MYH1B*, *MYH1F*, *MYH13*, *MYOCD*), adiposis and fibrosis (*PDGFRα*), extracellular matrix or sarcolemma composition (i.e., *COL6A3*, *FN1*, *SGCB*), muscle metabolism (*PNPLA7*), and human neuromuscular disorders (i.e., *FN1*, *COL6A3*, *SGCB, LRSAM1*).

**Table 4:** Set of genes selected for eQTL detection

Trait	Candidate gene	Name	Gene ID	GGA	Start^a^	End^a^
WS	*MYH15*	myosin heavy chain 15	395,534	1	87,501,148	87,547,719
WS	*PDGFRα*	platelet derived growth factor receptor alpha	395,509	4	65,808,218	65,842,682
WS	*SGCB*	sarcoglycan β	422,760	4	66,587,612	66,593,633
WS	*FN1*	fibronectin 1	396,133	7	4,389,536	4,439,464
WS	*COL6A3*	collagen type VI alpha 3 chain	396,548	7	4,808,221	4,861,524
WS	*LRSAM1*	leucine-rich repeat and sterile alpha motif containing 1	417,265	17	2,026,150	2,051,581
WS	*PNPLA7*	patatin-like phospholipase domain containing 7	427,774	17	2,080,356	2,196,812
WS	*TUBB4B*	tubulin beta 4B class IVb	417,255	17	2,368,202	2,370,299
WS	*MYH1E*	myosin heavy chain 1E	427,788	18	588,354	611,030
WS	*MYH1B*	myosin heavy chain 1B	374,069	18	633,783	651,055
WS	*MYH1F*	myosin heavy chain 1F	768,566	18	431,376	449,075
WS	*MYH13*	myosin heavy chain 13	8735	18	346,675	384,341
WS	*MYOCD*	myocardin	427,790	18	830,396	930,411
CL, DL, L*,b*	*DYSF*	dysferlin	425,353	4	91,211,949	91,279,004
CL, DL, L*,b*	*CAV3*	caveolin 3	378,796	12	19,314,666	19,318,095
CL, DL, L*,b*	*CAPN3*	calpain 3	423,233	5	25,645,486	25,673,883

^a^ Positions are indicated on galgal5 assembly

The *Myosin Heavy chain 15* gene (*MYH15*) located in the region of interest on GGA1 (Additional file 2: Figure S2) is involved in the contraction, development, and regeneration of avian skeletal muscles [27]. The most significant SNP associated with WS is located in the gene itself.

The *Platelet Derived Growth Factor Receptor Alpha* gene (*PDGFRα*), which is located upstream of the SNP of interest for WS and BMY on GGA4 (Additional file 3: Figure S3), is known to be a marker of fibro-adipogenic precursors (FAPs) recently characterized as playing a role in muscle regeneration and repair. Under pathological conditions like muscular dystrophy, FAPs differentiate into fibroblasts and adipocytes, leading to fat and connective tissue infiltration. In addition, FAPs promote myoblast differentiation in co-cultivation experiments [28]. The *Sarcoglycan Beta* gene (*SGCB*) is another gene of interest for the GGA4 region (Additional file 3: Figure S3) as it codes for the beta sarcoglycan protein, a member of the sarcoglycan complex located to the sarcolemma. This subcomplex of the dystrophin-associated glycoprotein complex (DAG complex) contributes to muscle tissue structure maintenance and stability and transfer of mechanical strength all along the sarcolemma during muscle contraction. Mutations in this gene were identified in human limb-girdle (LGMD1E) muscular dystrophy [29]. It has been shown that transgenic beta-sarcoglycan-deficient mice exhibited progressive muscular dystrophy with extensive degeneration and regeneration of muscle fibers [30]. These mice also exhibited muscular hypertrophy and whitish stripes within the muscles [30]. This makes *SGCB* an interesting positional and functional candidate gene for the QTL3 region, for which a pleiotropic effect on WS and BMY was suggested.

The *Fibronectin 1* gene (*FN1*) is the closest gene to the WS QTL region on GGA7 (Additional file 4: Figure S4) and encodes a glycoprotein, which contributes to the creation of fibers and extracellular matrix during tissue repair after degeneration. In addition, an increase in fibronectin levels is a biomarker of Duchenne muscular dystrophy [31]. The *Collagen type VI Alpha 3-chain* gene (*COL6A3*) is another interesting gene for this region (Additional file 4: Figure S4). It produces collagen molecules found in the extracellular matrix and surrounding cells that make up the muscles used for movement. Mutations in this gene are associated with Bethlem myopathy and Ullrich congenital myopathy [32].

One candidate gene in the region of interest on GGA17 (Additional file 5: Figure S5) is the *Leucine-rich Repeat and Sterile Alpha Motif containing 1* gene (*LRSAM1*). It encodes an ubiquitin-protein ligase with a role in sorting internalized cell-surface receptor proteins. Mutations in the *LRSAM1* gene have been shown to cause an axonal form of Charcot-Marie-Tooth hereditary neuropathy (CMT2P), characterized by progressive muscle weakness and atrophy [33]. A second candidate gene*, Patatin-like Phospholipase domain containing 7* gene (*PNPLA7*), is an insulin-regulated lysophospholipase expressed in muscle and fat. The regulation of its expression by nutritional status and insulin suggests a role in the catabolism of lipid precursors and/or mediators that affect energy metabolism in mammals [34]. Furthermore, another member of the patatin family, *PNPLA2*, is involved in neutral lipid storage disease associated with myopathy that is characterized by the accumulation of triglycerides in multiple tissues [35]. The last candidate gene of this region, *Tubulin Beta-4B chain* (*TUBB4B*), codes for a protein (tubulin) which is the major constituent of microtubules. Microtubules are major constituents of the cytoskeleton and have major roles in maintenance of the cell structure and intracellular transport.

Finally, in addition to *MYH15* on GGA1, four fast myosin heavy chain isoforms (i.e. *MYH1E*, *MYH1B*, *MYH1F*, and *MYH13)* and myocardin (*MYOCD)* were selected. As shown in Additional file 6: Figure S6, these genes are grouped within a cluster located at the beginning of GGA18 (0.35 to 0.93 Mb), near the QTL region associated with WS (2.77 Mb). Out of all the groups of myosins, we chose to focus on them because they are fairly well annotated. Furthermore, a previous histological study on the same genetic lines highlighted a greater expression of embryonic (*MYH1B*) and neonatal (*MYH1F*) myosin heavy chain isoforms and a lower expression of adult myosin heavy chain (*MYH1E*) in the pHu + than in the pHu- muscle, which is consistent with a stronger muscle fiber regeneration process [36]. In addition, a transcriptomic study on the same model showed differential expression of *MYH13* and *MYOCD* genes between normal and WS fillets [37].

#### Other meat quality phenotypes

Because of its high level of significance and pleiotropic effect on CL, DL, L* and b*, we looked for candidate genes within the QTL4 region (Table 2). Visualization of the gene environment showed that the *dysferlin* (*DYSF*) gene (91.21–91.27 Mb) is close to the QTL region (90.82 to 91.20 Mb) (Additional file 7: Figure S7). The *dysferlin* gene encodes a protein that is found in the sarcolemma. It is involved in sarcolemma repair and some results suggest that dysferlin may also be involved in the formation of new muscle fibers (regeneration) and inflammation [38]. Furthermore, dysferlin was identified as a gene that is mutated in limb-girdle muscular dystrophy and some congenital myopathies. Some studies demonstrated that dysferlin-null mice developed progressive muscular dystrophy caused by failure of plasma membrane repair in muscle [38, 39]. Two genes encoding for skeletal muscle proteins (i.e. *caveolin 3* and *calpain 3*) were added to the set of genes of interest both for their interaction with the *dysferlin* gene [40, 41], and their involvement in human limb-girdle muscular dystrophies (LGMD1C and LGMD2A, respectively) [42, 43]. *Caveolin 3* (*CAV3*) may play a key role in the fusion of myoblasts into myotubes during the maturation process of muscle fibers. *Calpain 3* (*CAPN3*) is an intracellular protease specific to the muscle, involved in the calcium-dependent proteolytic system. They are known to catalyze the limited proteolysis of proteins (i.e., desmin and vimentin) involved in cytoskeletal modelling or signal transduction and to play a role in regeneration processes [44, 45].

Of the 16 candidate genes considered in the study, whether for WS or meat quality phenotypes, more than half are components of the extracellular matrix, sarcolemma, or muscles. Interestingly, several of them (i.e., *COL6A3*, *CAV3*, *CAPN3*, *DYSF*, *SGCB*) are associated with, or are members of the dystrophin complex. This complex contains dystrophin, which is at the origin of the genetic defect causing Duchenne muscular dystrophy (DMD) in humans, and establishes a direct link between the actin cytoskeleton and the extracellular matrix [46]. Thus, it participates in maintaining the extracellular matrix and sarcolemma. A perturbation within this complex or these associated proteins, such as a loss of the mechanical link between the cytoskeleton, extracellular matrix, and sarcolemma, may result in muscle fragility, contraction-induced damage, and necrosis [47].

### eQTL detection

GWAS can be applied in order to map eQTLs that control the transcript level of genes. Co-localization between phenotypic QTLs and eQTLs is particularly useful in order to identify candidate genes, which are potentially causative [48]. In the current study, the expression of all identified candidate genes was quantified using RT-qPCR. After GWAS analysis, 132 SNPs were found to be significantly associated with molecular phenotypes including 24 SNPs significant at the genome threshold (Additional file 8: Table S1). They defined 21 eQTL regions located on 16 chromosomes.

eQTLs are categorized as *cis* or *trans*, where *cis* eQTLs represent a polymorphism physically located near the gene itself, for example, a promoter polymorphism that gives rise to differential expression of the gene [48]. In the current study, we defined as *cis* eQTLs, SNPs that were within 1 Mb of the annotated start or stop site of the corresponding structural gene. eQTLs that did not fulfill this condition were considered to be *trans* eQTLs. These regions could, for example, contain a polymorphism in a transcription factor that correspondingly modulates the level of transcripts for target genes [48]. Of the 21 eQTL regions, three were *cis* eQTLs and 18 were *trans* eQTLs.

#### Cis eQTLs

**Fig. 3:**
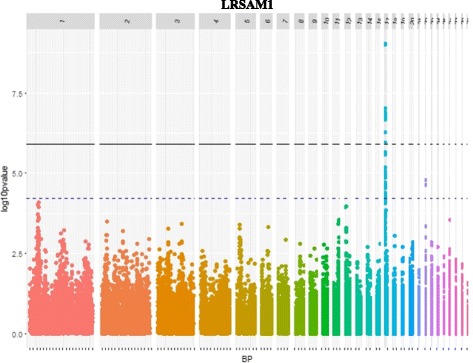
Manhattan plot showing the association of SNPs with *LRSAM1* expression. Black line represents the 5% genome-wide threshold and blue line the 5% GGA17-wide threshold

The first *cis* eQTL relates to *LRSAM1* gene. Out of 31 significant SNPs associated with its expression, 28 were located in a region close to the gene (eQTL15) and one was in the gene itself (Gga_rs15034052 at 2.04 Mb). Eleven out of these 29 SNPs were significant at the genome threshold (Fig. 3, Additional file 8: Table S1).

The second *cis* eQTL relates to *MYH1F* gene. Of the 16 SNPs significantly associated with its expression, two were significant at the genome threshold on GGA18 (Additional file 9: Figure S8, Additional file 8: Table S1). The expression of *MYH1F* is associated with 14 SNPs, which are close to the gene (eQTL16) and located within a region containing the cluster of myosin heavy chain isoforms.

The last *cis* eQTL relates to *CAV3* gene. GWAS analysis identified 29 SNPs significantly associated with gene expression and which defined two eQTL regions, one *cis* eQTL on GGA12 (eQTL13) (Additional file 10: Figure S9) and one *trans* eQTL on GGA18 (eQTL17) (Additional file 8: Table S1). In contrast to the two other *cis* eQTLs, the *CAV3* cis eQTL was significant only at the chromosome level.

#### Trans eQTLs

**Fig. 4:**
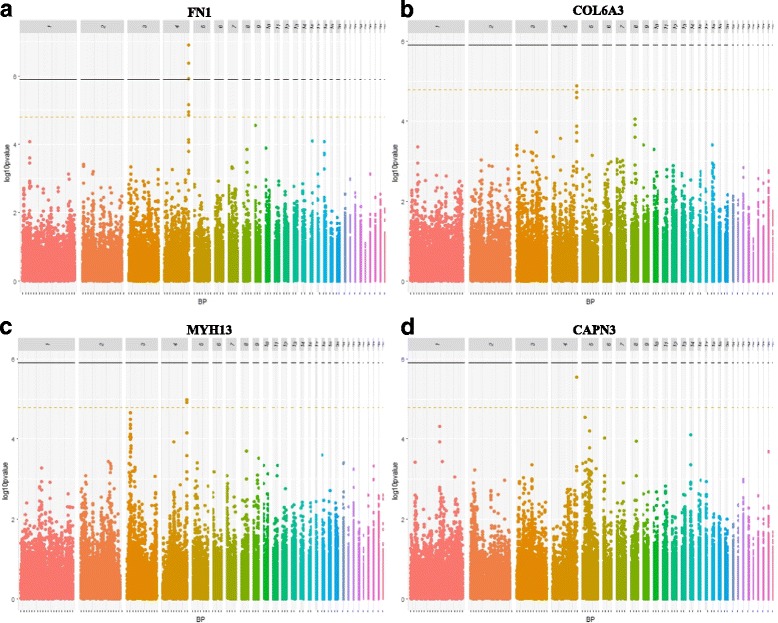
Manhattan plot showing the association of SNPs with *FN1* (**a**), *COL6A3* (**b**), *MYH13* (**c**) and *CAPN3* (**d**) expressions. Black line represents the 5% genome-wide threshold and orange line the 5% GGA4-wide threshold

Among all *trans* eQTLs (Additional file 8: Table S1), four regions seem to control several molecular phenotypes. The first one is on GGA2 (eQTL3), where a single SNP is associated with the expression of *PNPLA7* and *DYSF* genes (Gga_rs14139566 at 12.55 Mb). The second, on GGA6 (eQTL9), includes several SNPs for the *MYH1B* gene, and one single SNP for *MYH1B*, *DYSF* and *PDGFRα* (Gga_rs14581613 at 2.03 Mb). The third region is located on GGA22 (eQTL18) where two single SNPs are identified (GGaluGA186934 at 2.90 Mb and GGaluGA186952 at 2.94 Mb), for *LRSAM1*, *PNPLA7* and *TUBB4B* gene expression. Finally, one last region appears to control several molecular phenotypes on GGA4 (eQTL6). This region is associated with the expression of *FN1*, *MYH13*, *COL6A3,* and *CAPN3*. Of the six SNPs significantly associated with these molecular phenotypes, half are associated with at least two gene expressions: one single SNP between *FN1* and *MYH13* (Gga_rs16454745 at 91.10 Mb), another between *FN1* and *COL6A3* (Gga_rs14506759 at 91.11 Mb), and finally a last between the four genes (Gga_rs15648179 and 91.20 Mb) (Fig. 4). This last region seems particularly interesting since it regulates the expression of three genes involved in human muscle diseases, as a biological marker (*FN1*) or genetic markers (*COL6A3* and *CAPN3*).

### Co-localization between QTLs and eQTLs

Interestingly, several co-localizations between QTL and eQTL regions were observed on GGA4, GGA5, and GGGA17, which could suggest causative genes and gene networks involved in the variability of meat quality traits or breast meat yield.

#### White striping

The first co-localization concerns the region of GGA17 associated with both the expression of *LRSAM1* gene and WS defect (eQTL15 and QTL12) (Additional file 8: Table S1, Additional file 11: Table S2). The level of significance was much higher for the molecular phenotype than for the WS phenotype, which suggested a tighter regulation of the gene expression by this region. Although three genes of interest (*LRSAM1*, *PNPLA7*, *TUBB4B*) were tested for this region, only *LRSAM1* was significantly regulated and appeared to be a putative causative gene. In addition to being a positional and expressional candidate gene, *LRSAM1* is also a functional candidate gene, since a mutation in this gene results in a form of hereditary motor and sensory neuropathy where slowly progressive distal muscle weakness and atrophy were observed [33].

A *cis* eQTL for the neonatal myosin heavy chain form *MYH1F* (0.30 to 0.83 Mb) was also observed near the QTL associated with WS on the region of interest on GGA18 (2.77 Mb). Embryonic (*MYH1B*) and neonatal (*MYH1F*) forms of myosin heavy chain are found in breast muscle during its early development or in muscle fibers regeneration. A characterization of the muscle fibers was previously performed in the pHu + and pHu- lines. The muscle fibers of the pHu + birds were characterized by a higher number of cells expressing the developmental embryonic and neonatal isoforms of the fast myosin heavy chain [36], as well as a higher incidence of WS than was found in pHu- muscle fibers [19]. *MYH1F* could be an interesting molecular marker of WS in relation to the regeneration process. However, we cannot conclude that there is a single mutation affecting this molecular phenotype and WS, since the positions of the QTL and eQTL were rather distant.

#### Body composition and meat quality traits

A first co-localization was found on GGA5 between PMY QTL (QTL5) and *SGCB*-eQTL (eQTL7) (Additional file 8: Table S1, Additional file 11: Table S2). As previously reported, mutations within this gene result in a loss or a large decrease in the sarcoglycan complex [30]. Mice carrying the mutation are hypertrophic and larger than wild-type phenotypes. No significant SNP for WS was found in this region in the current study, which did not support any role of the region in the genetic relationship observed between PMY and WS in this population [19]. Nevertheless, our results indicated that *SGCB* could be an interesting molecular actor to consider in future studies on the control of muscle development and integrity.

Another co-localization was found on GGA4, between the previously identified pleiotropic region (QTL4) containing the SNPs strongly associated with CL, DL, and color parameters (L* and b*), and the eQTL region controlling *FN1*, *COL6A3*, *MYH13,* and *CAPN3* expression (eQTL6). This region seems to regulate meat quality phenotypes possibly related to muscle cell integrity and expression of genes involved in human neuromuscular disorders, as well as in the structure and composition of muscle fibers.

The QTL mapping revealed several regions of importance for the control of meat quality phenotypes. The eQTL analysis suggested some candidate genes and molecular networks associated with WS and meat quality traits. On the other hand, the eQTL analysis for *MYH15*, S*GCB,* and *DYSF* did not evidence any co-localizations with WS nor with meat quality traits. However, we cannot exclude an effect of these genes. They remain interesting candidate genes whose action could be modulated by mutations in the coding region, leading to variations in protein stability or enzymatic activity, or by post-translation modification [48]. This was the case for the *RN* gene (Rendement Napole) in pigs where a loss of function mutation in the *PRKAG3* gene is associated with an excess of glycogen content in the skeletal muscles [49]; or for the *MSTN* gene (myostatin), for which a loss of function mutation results in muscular hypertrophy in cattle and sheep [50]. Further analyses are needed to elucidate the role of these genes in the initiation of biological processes involved in the appearance of WS defect and more generally in poultry meat quality variations.

## Conclusions

This study reported the first QTLs for WS in chicken. It did not support the existence of a major gene, but was in favor of a polygenic inheritance of the defect and of the other studied meat quality traits. We identified several candidate genes involved in muscle metabolism and structure, and also in neuromuscular disorders for some of them. The eQTL analyses confirmed that they were part of molecular networks associated with WS and meat quality phenotypes and suggested a few putative causative genes. This study provides a first set of molecular and genetic markers for WS that will have to be validated and enriched by the study of additional genetic populations and the integration of molecular and genetic information.

## Methods

### Birds and housing

Meat pHu reflects the level of muscle glycogen content and is a determining criterion of meat quality. In this study we used two broiler lines divergently selected for *Pectoralis major* pHu according to a breeding scheme described in Alnahhas et al. [25] and which exhibit WS defect.

WS, body composition, and meat quality traits were evaluated in 558 birds (253 males and 305 females) from the 6th generation of divergent selection (G6). Of this total, 278 broilers (135 males and 143 females) were from the pHu + line (selected for high breast pHu value) and 280 broilers (118 males and 162 females) from the pHu- line (selected for low breast pHu value). Birds were reared as a single population (males and females from both lines), following standard rearing practices and had ad libitum access to feed and water. They were produced in two successive batches and slaughtered at 6 weeks of age at the PEAT experimental unit (INRA, Centre Val de Loire, Nouzilly, France). After hanging the birds on the processing chain, they were automatically stunned in a water bath using a constant electric current and slaughtered manually by cutting the carotid artery and the jugular vein at the ventral surface of the neck from inside the oral cavity. Samples of *P. major* were collected fifteen minutes after slaughter, snap-frozen in liquid nitrogen and stored at − 80 °C until further analysis. Breasts were visually graded a day after slaughter by one trained person. The categories of WS were defined according to a modified version of the scale of Kuttappan et al. [14] to account for the lower degree of severity in our experimental population (0 = normal, absence of WS, 1 = moderate white striping, corresponding to striation thickness ≤ 1 mm, and 2 = severe occurrence of white striping, corresponding to striation thickness ≥ 1 mm). Body composition was characterized for all birds through the measurement of BMY, abdominal fat percentage (AFP), PMY*,* PmY and thigh yield (TY) expressed in relation to body weight (BW). Breast meat quality was evaluated on *Pectoralis major* muscle through the measurement of color parameters (L*, a*, b*), CL, DL, Warner-Bratzler shear force (SF) of cooked meat, curing-cooking yield (CCY), IMF, and TBA-RS index. All these measurements were realized as described in Alnahhas et al. [19].

### Genotyping

The 558 birds were genotyped at Labogena Laboratory (Jouy en Josas, France) using the Illumina chicken SNP 57 K Beadchip containing 57,636 SNPs. Quality control of the genotype data was performed using PLINK 1.9 and included animal call rate (> 95%), SNP call rate (> 95%), and minor allele frequency (> 5%). Finally, after filters, all animals were kept and 40,195 out of 57,636 SNPs distributed along 28 autosomal chromosomes were validated for further analyses.

### Gene expression

Gene expression was determined by RT-qPCR performed on one batch of animals from the 6th generation of divergent selection (145 pHu+, 136 pHu-), as described by Beauclercq et al. [36]. Total RNA was extracted from *Pectoralis major* muscle sampled for each individual, using RNA NOW (Ozyme, St Quentin en Yvelines, France). Ten μg of RNA from each sample were reverse-transcribed using RNase H^−^MMLV reverse transcriptase (Superscript II, Invitrogen, Illkirch, France) and random primers (Promega, Charbonnières les Bains, France). Primers targeting the studied genes were designed with Primer3 version 4.0.0 [51, 52]. The list of primer sequences is available in Additional file 11: Table S2. Their products of amplification were analyzed by electrophoresis and further sequenced.

The level of mRNA expression of candidate genes was quantified using a Fluidigm Biomark microfluidic device (Fluidigm, South San Francisco, CA, USA) according to the manufacturer’s protocol (ADP37). Because *MYH15* and *PDGFRα* genes were added to the list of candidate genes later, their expression was quantified by RT-qPCR using a Roche LightCycler® 480 II (Roche Applied Science, Penzberg, Upper Bavaria, Germany) and the Takyon® (Eurogentec, Liege, Belgium), according to the manufacturer’s recommendations. Quantitative PCR conditions were set at 95 °C for 5 min, followed by forty-five cycles of 10 s at 95 °C, 20 s at 60 °C and 10 s at 72 °C. Two references were used: *PDE3B* (phosphodiesterase 3B), which is invariant whatever the sex and degree of severity of WS within and between the lines, was used as the housekeeping gene to normalize the Ct values; and a mix of chicken *Pectoralis major* muscle cDNA. The calculation of absolute mRNA levels was based on the PCR efficiency and the threshold cycle (CT) deviation of an unknown cDNA versus the control cDNA according to the equation proposed by Pfaffl [53]. The normalized Ct values (ΔΔCt) were used as the molecular phenotype for the GWAS analyses.

### QTL and eQTL detections

Genome-wide association studies were performed on classical and molecular phenotypes using an univariate linear mixed model (LMM) implemented in GEMMA (Genome-wide Efficient Mixed Model Association) software [54]. The model used for analyses was:

$$ \mathrm{y}=\mathrm{W}\alpha +\mathrm{x}\beta +\mathrm{u}+\upepsilon, $$

where **y** is a n-vector of phenotypes for n individuals, **W** the n x c matrix of covariates which contains fixed effects of sex and batch (only sex effect for eQTL detection) and including a column of 1 s for the general mean, **α** the c-vector of the corresponding coefficients including the intercept, **x** the n-vector of marker genotypes, **β** a p-vector of marker effects, **u** the n vector of polygenic effects, and **ϵ** the n-vector of the residuals. **u** follows a multivariate normal distribution: **u** ~ MVN_n_(0, λτ^− 1^**K**) where τ^− 1^ is the variance of the residual errors, λ is the ratio between the genetic variance (variance explained by the SNPs) and the residual variance, and **K** is a known n x n relationship matrix estimated from genotypes, using a n x p matrix of genotypes (**X**):

$$ \mathrm{K}=\frac{1}{\mathrm{p}}\sum \limits_{i=1}^{\mathrm{p}}\left({\mathrm{x}}_i-{1}_{\mathrm{n}}{\overline{x}}_i\right){\left({\mathrm{x}}_i-{1}_{\mathrm{n}}{\overline{x}}_{\mathrm{i}}\right)}^{\mathrm{T}.} $$

This expression was used to calculate **K** where x_*i*_ is the *i*th column representing genotypes of *i*th SNP, ${\overline{x}}_i$ is the sample mean and 1_n_ is a n × 1 vector of 1’s. **ϵ** follows a multivariate normal distribution: **ϵ** ~ MVN_n_(0,τ^−1^**I**_**n**_) where **I**_**n**_ is an n x n identity matrix.

To control the family-wise error rate (FWER) in the context of multiple hypothesis testing, a Bonferroni correction at 0.05 significant level was applied for both genome-wide and chromosome-wide thresholds (significant level = α/number of SNPs). Any SNP with a *P*-value < 1.21 × 10^− 6^ was considered to be significantly associated at the genome-wide threshold. The chromosome-wide significance thresholds ranged from 7.56 × 10^− 6^ to 3.36 × 10^− 4^, depending on the chromosome size.

## Additional files

Additional file 1: **Figure S1.** Manhattan plot showing the association of SNPs with WS in the pHu + (a) and the pHu- (b) line. Black line represents the 5% genome-wide threshold, red line the 5% GGA1-wide threshold, blue line the 5% GGA17-wide threshold and purple line the 5% GGA20-wide threshold. (DOCX 1204 kb)

Additional file 2: **Figure S2.** Gene environment of the region of interest for WS on GGA1. The first track corresponds to the chromosome 1 where the region of interest is indicated by a red line. The second track corresponds to the genomic axis for the region of interest. The third track corresponds to the gene model based on GalGal5 assembly. The last track corresponds to the Manhattan plot of the region of interest where the orange line represents the chromosome threshold and the most significant SNP for WS is highlighted in red. (PDF 8 kb)

Additional file 3: **Figure S3.** Gene environment of the region of interest for WS and BMY on GGA4. The first track corresponds to the chromosome 4 where the region of interest is indicated by a red box. The second track corresponds to the genomic axis for the region of interest. The third track corresponds to the gene model based on GalGal5 assembly. The two last tracks correspond to the Manhattan plot of BMY and WS respectively, on the region of interest, where the orange line represents the chromosome threshold and the most significant SNP for WS and BMY is highlighted in red. (PDF 10 kb)

Additional file 4: **Figure S4.** Gene environment of the region of interest for WS on GGA7. The first track corresponds to the chromosome 7 where the region of interest is indicated by a red box. The second track corresponds to the genomic axis for the region of interest. The third track corresponds to the gene model based on GalGal5 assembly. The last track corresponds to the Manhattan plot of the region of interest where the orange line represents the chromosome threshold and the most significant SNP is highlighted in red. (PDF 8 kb)

Additional file 5: **Figure S5.** Gene environment of the region of interest for WS on GGA17. The first track corresponds to the chromosome 17 where the region of interest is indicated by a red box. The second track corresponds to the genomic axis for the region of interest. The third track corresponds to the gene model based on GalGal5 assembly. The last track corresponds to the Manhattan plot of the region of interest where the orange line represents the chromosome threshold and the most significant SNP is highlighted in red. (PDF 9 kb)

Additional file 6: **Figure S6.** Gene environment of the region of interest for WS on GGA18. The first track corresponds to the chromosome 18 where the region of interest is indicated by a red box. The second track corresponds to the genomic axis for the region of interest. The third track corresponds to the gene model based on GalGal5 assembly. The last track corresponds to the Manhattan plot of the region of interest where the orange line represents the chromosome threshold and the most significant SNP is highlighted in red. (PDF 10 kb)

Additional file 7: **Figure S7.** Gene environment of the pleiotropic region on GGA4. The first track corresponds to the chromosome 4 where the region of interest is indicated by a red box. The second track corresponds to the genomic axis for the region of interest. The third track corresponds to the gene model based on GalGal5 assembly. The last track corresponds to the Manhattan plot of CL, which has the most significant SNP in the pleiotropic region. Orange line represents the chromosome threshold and the most significant SNP is highlighted in red. (PDF 9 kb)

Additional file 8: **Table S1.** Significant SNPs for the level of expression of the 16 candidate genes. (DOCX 36 kb)

Additional file 9: **Figure S8.** Manhattan plot showing the association of SNPs with *MYH1F* expression. Black line represents the 5% genome-wide threshold and blue line the 5% GGA18-wide threshold. (DOCX 560 kb)

Additional file 10: **Figure S9.** Manhattan plot showing the association of SNPs with *CAV3* expression. Black line represents the 5% genome-wide threshold and green line the 5% GGA12-wide threshold. (DOCX 585 kb)

Additional file 11: **Table S2.** Primer sequences of the 16 candidate genes and the housekeeping gene (PDE3B). (DOCX 14 kb)
