# Supplementary figures and images for: Mapping QTL for white striping in relation to breast muscle yield and meat quality traits in broiler chickens
Source: BMC Genomics. 2018 Mar 20;19:202. doi: 10.1186/s12864-018-4598-9 (PMC5859760; doi:10.1186/s12864-018-4598-9)

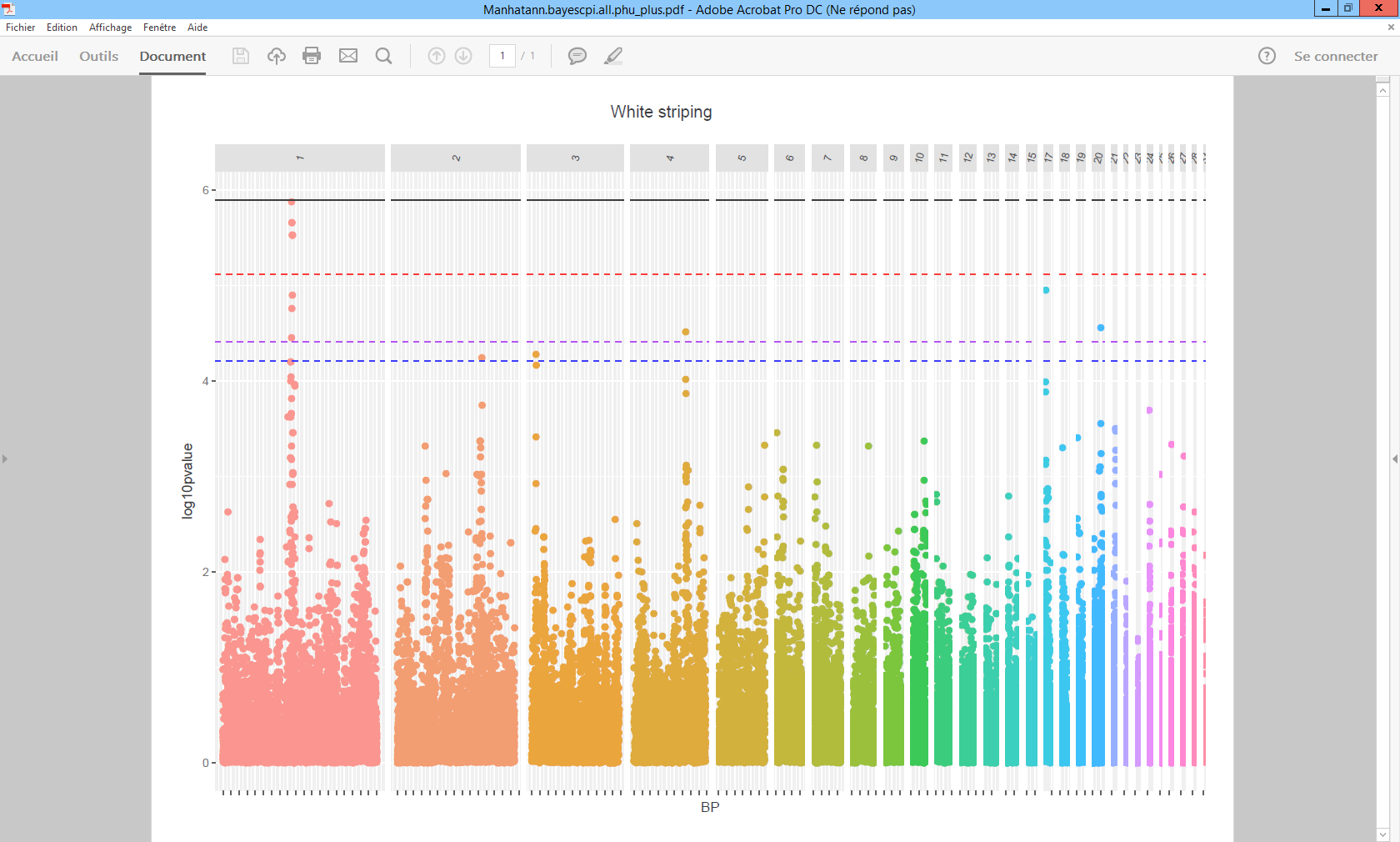


**(a)**

**White striping**

**pHu + line**


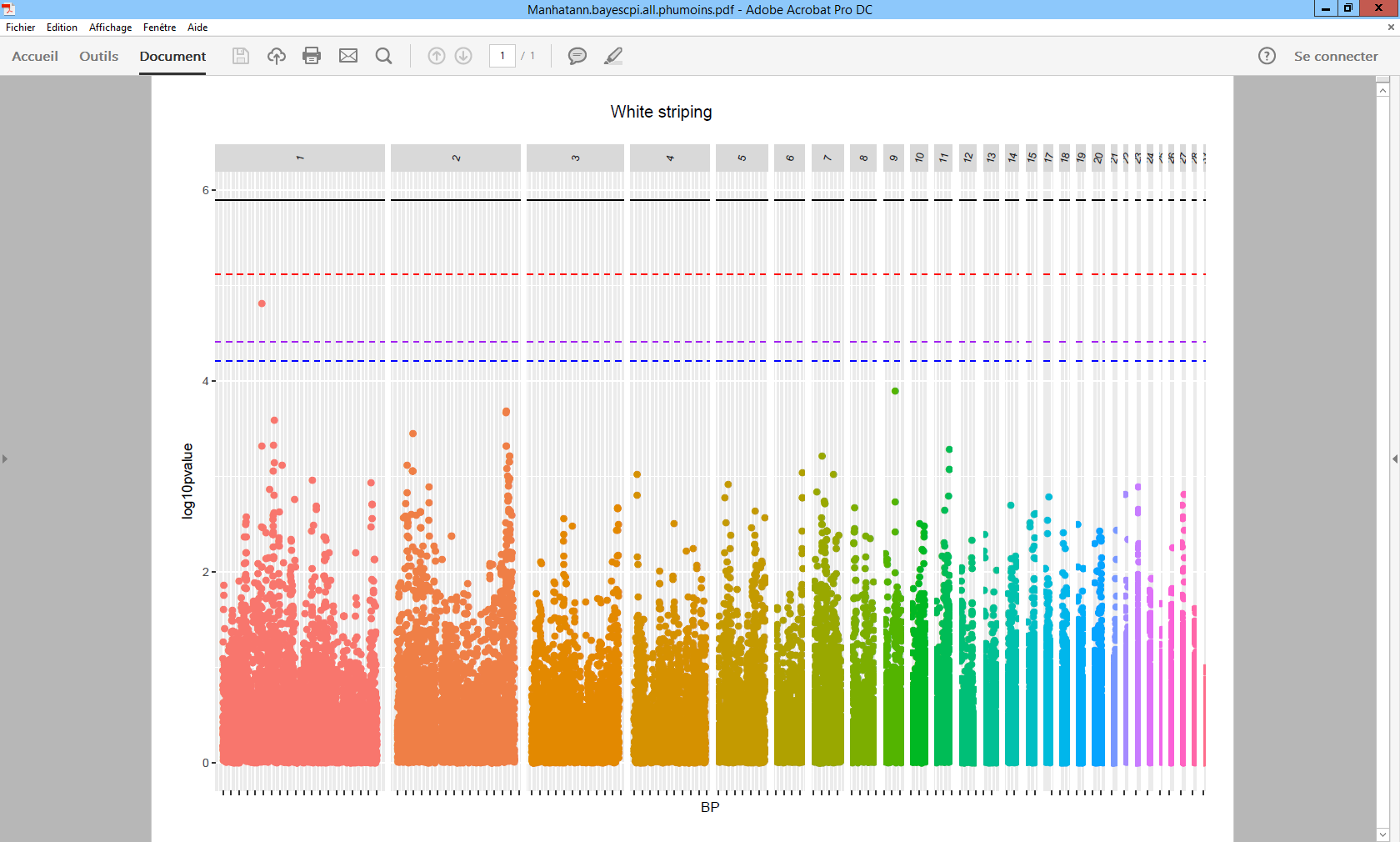


**(b)**

**White striping**

**pHu – line**

Supplement: Supplementary file 1 — Figure S1. Manhattan plot showing the association of SNPs with WS in the pHu + (a) and the pHu- (b) line. Black line represents the 5% genome-wide threshold, red line the 5% GGA1-wide threshold, blue line the 5% GGA17-wide threshold and purple line the 5% GGA20-wide threshold. (DOCX 1204 kb) [file 12864_2018_4598_MOESM1_ESM.docx]

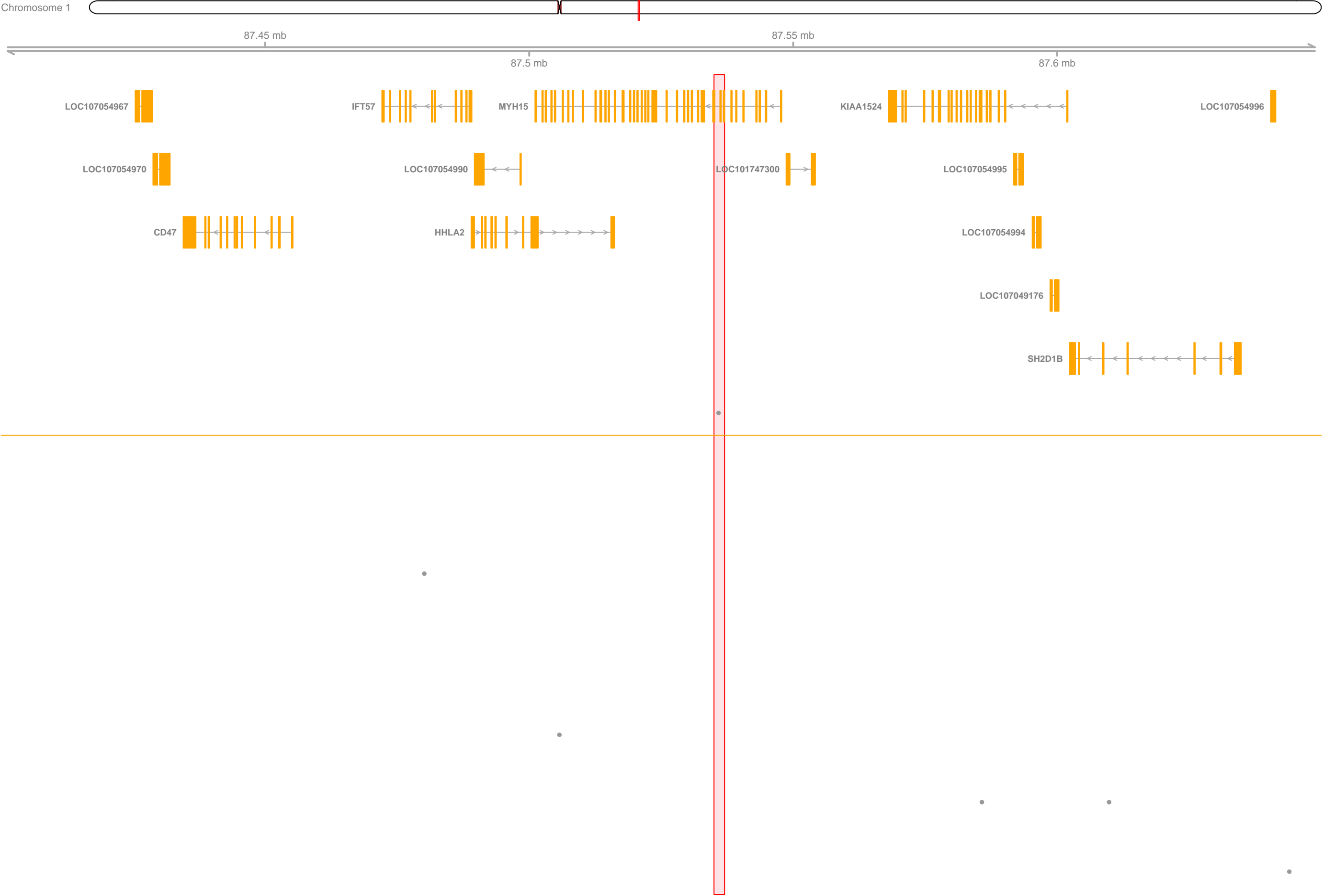

Supplement: Supplementary file 2 — Figure S2. Gene environment of the region of interest for WS on GGA1. The first track corresponds to the chromosome 1 where the region of interest is indicated by a red line. The second track corresponds to the genomic axis for the region of interest. The third track corresponds to the gene model based on GalGal5 assembly. The last track corresponds to the Manhattan plot of the region of interest where the orange line represents the chromosome threshold and the most significant SNP for WS is highlighted in red. (PDF 8 kb) [file 12864_2018_4598_MOESM2_ESM.pdf]

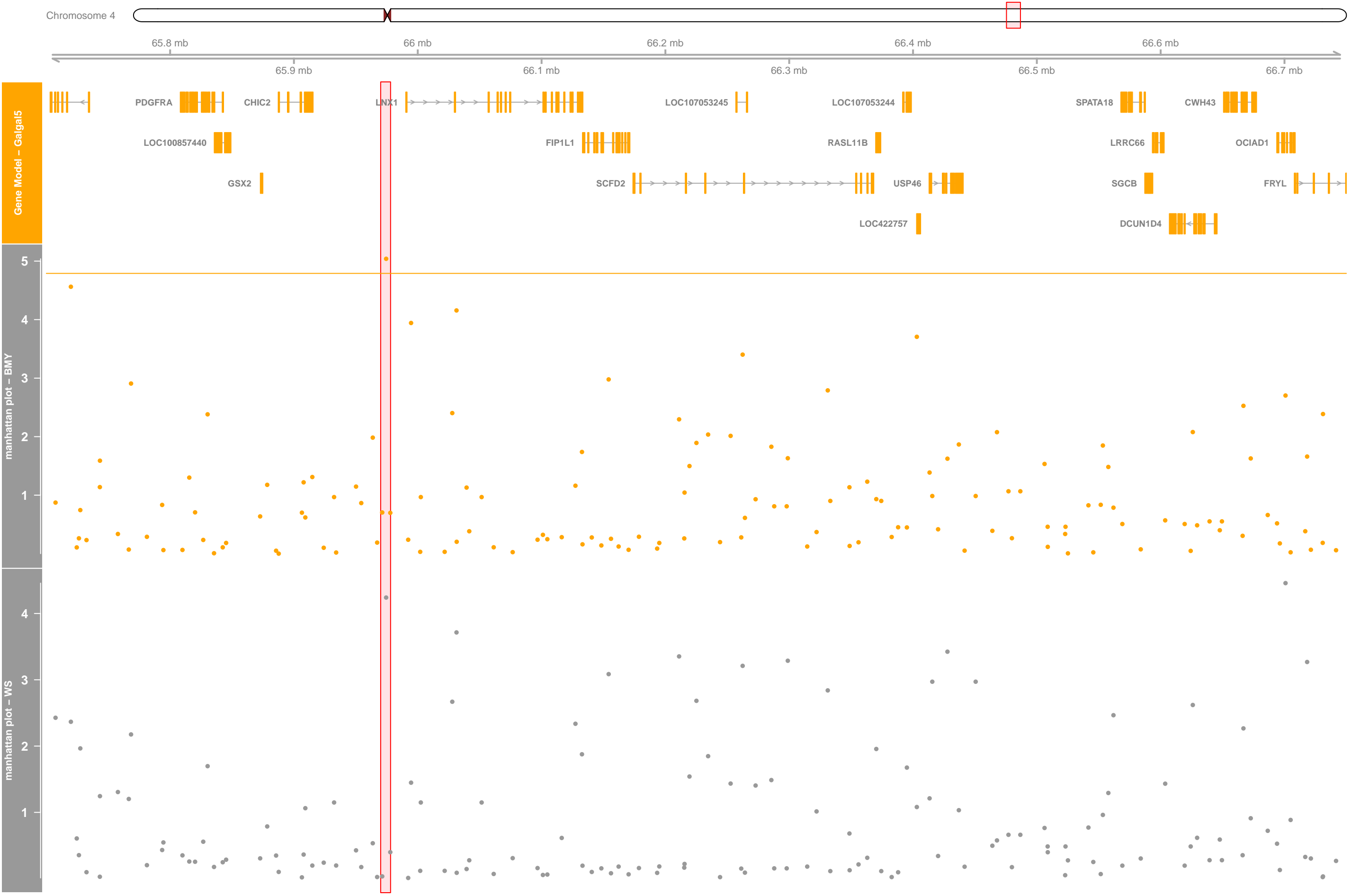

Supplement: Supplementary file 3 — Figure S3. Gene environment of the region of interest for WS and BMY on GGA4. The first track corresponds to the chromosome 4 where the region of interest is indicated by a red box. The second track corresponds to the genomic axis for the region of interest. The third track corresponds to the gene model based on GalGal5 assembly. The two last tracks correspond to the Manhattan plot of BMY and WS respectively, on the region of interest, where the orange line represents the chromosome threshold and the most significant SNP for WS and BMY is highlighted in red. (PDF 10 kb) [file 12864_2018_4598_MOESM3_ESM.pdf]

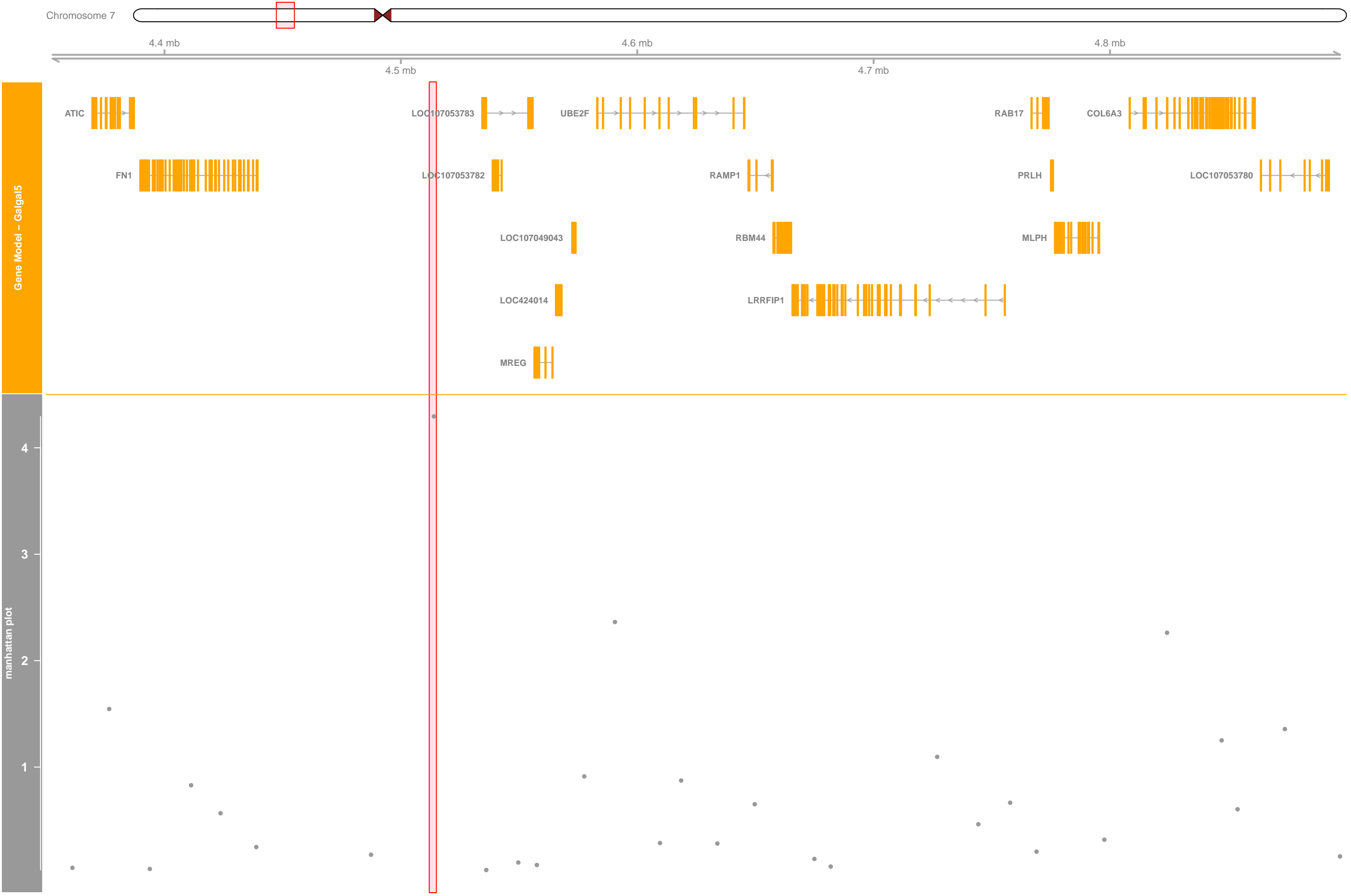

Supplement: Supplementary file 4 — Figure S4. Gene environment of the region of interest for WS on GGA7. The first track corresponds to the chromosome 7 where the region of interest is indicated by a red box. The second track corresponds to the genomic axis for the region of interest. The third track corresponds to the gene model based on GalGal5 assembly. The last track corresponds to the Manhattan plot of the region of interest where the orange line represents the chromosome threshold and the most significant SNP is highlighted in red. (PDF 8 kb) [file 12864_2018_4598_MOESM4_ESM.pdf]

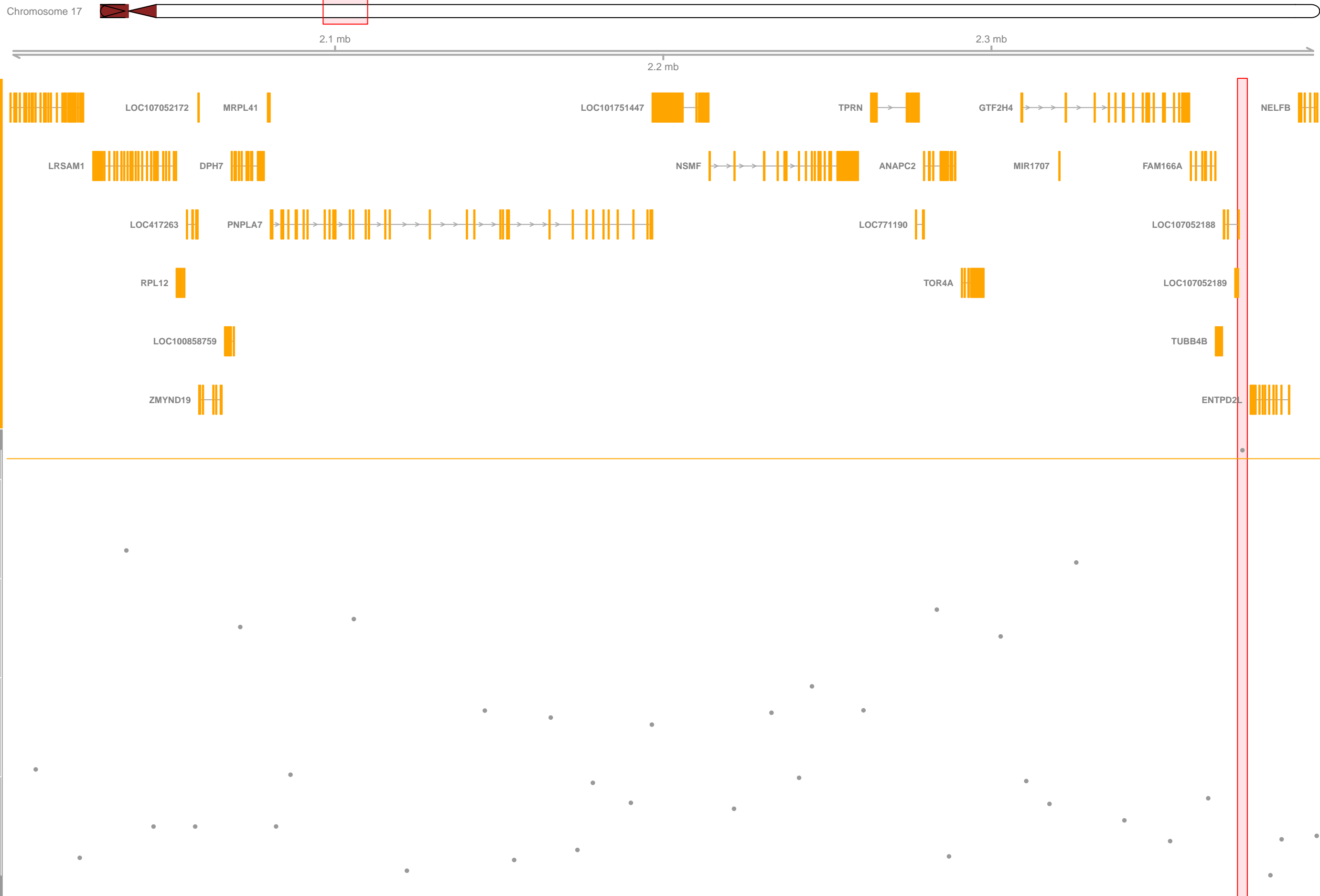

Supplement: Supplementary file 5 — Figure S5. Gene environment of the region of interest for WS on GGA17. The first track corresponds to the chromosome 17 where the region of interest is indicated by a red box. The second track corresponds to the genomic axis for the region of interest. The third track corresponds to the gene model based on GalGal5 assembly. The last track corresponds to the Manhattan plot of the region of interest where the orange line represents the chromosome threshold and the most significant SNP is highlighted in red. (PDF 9 kb) [file 12864_2018_4598_MOESM5_ESM.pdf]

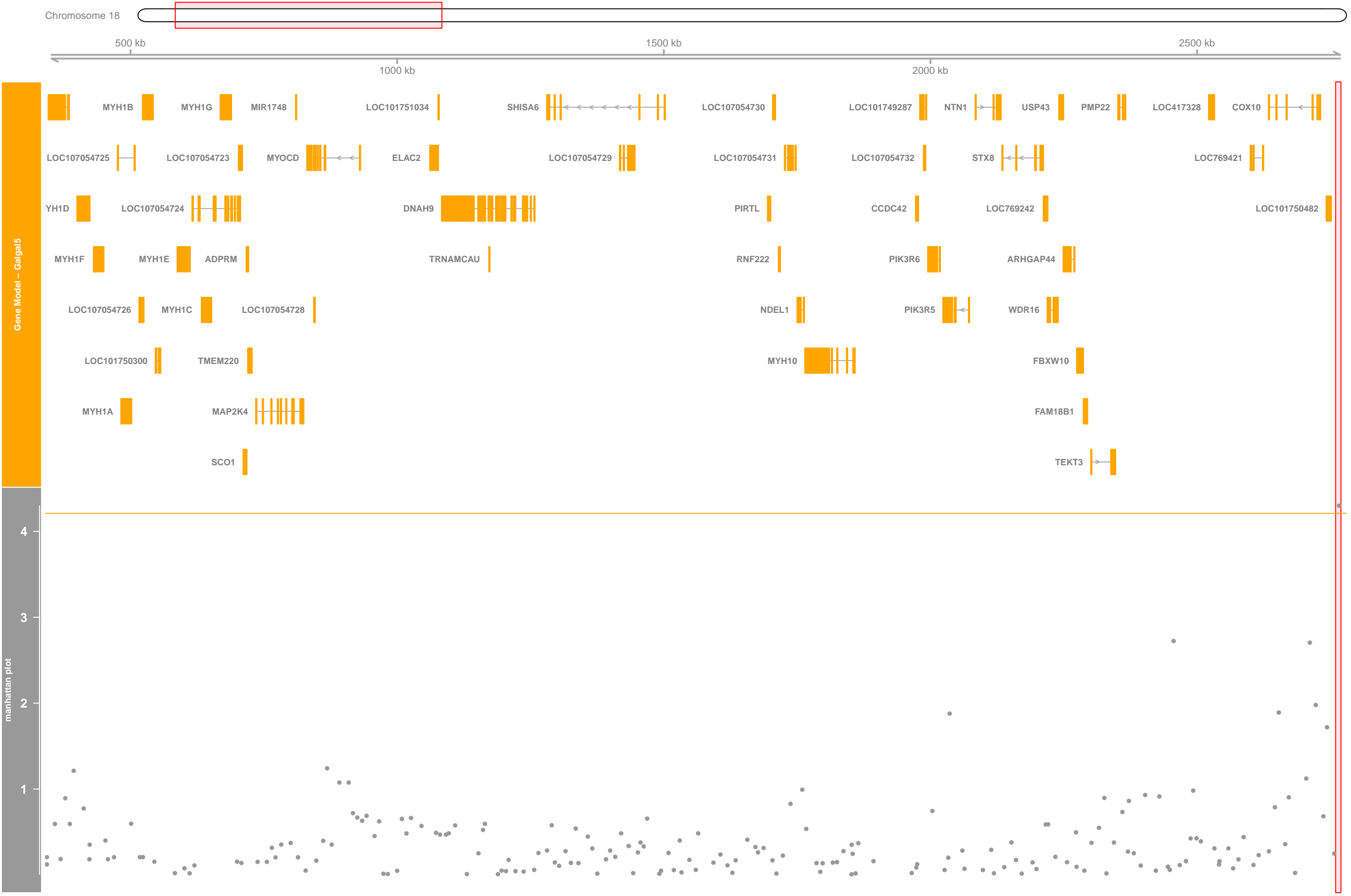

Supplement: Supplementary file 6 — Figure S6. Gene environment of the region of interest for WS on GGA18. The first track corresponds to the chromosome 18 where the region of interest is indicated by a red box. The second track corresponds to the genomic axis for the region of interest. The third track corresponds to the gene model based on GalGal5 assembly. The last track corresponds to the Manhattan plot of the region of interest where the orange line represents the chromosome threshold and the most significant SNP is highlighted in red. (PDF 10 kb) [file 12864_2018_4598_MOESM6_ESM.pdf]

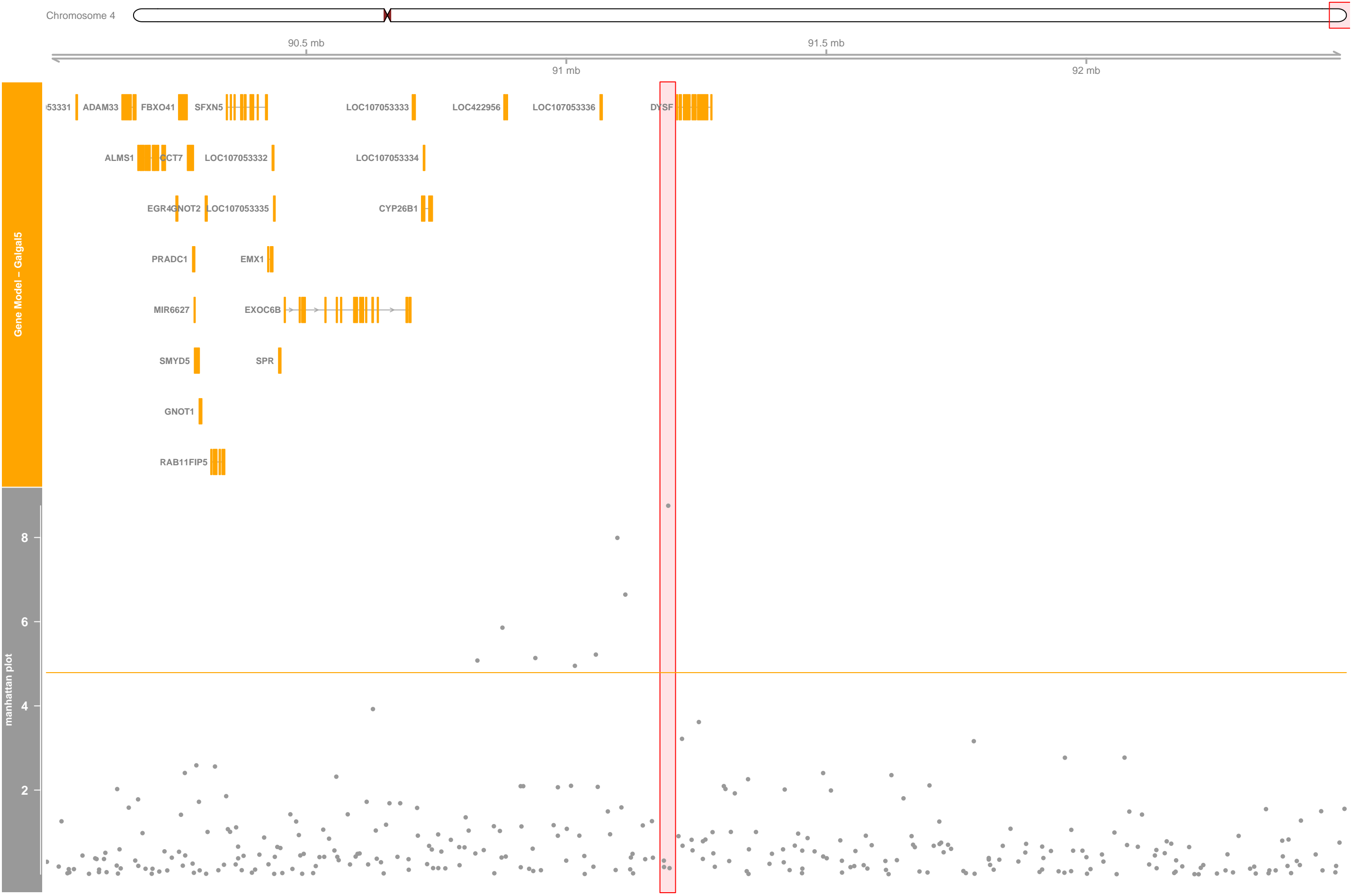

Supplement: Supplementary file 7 — Figure S7. Gene environment of the pleiotropic region on GGA4. The first track corresponds to the chromosome 4 where the region of interest is indicated by a red box. The second track corresponds to the genomic axis for the region of interest. The third track corresponds to the gene model based on GalGal5 assembly. The last track corresponds to the Manhattan plot of CL, which has the most significant SNP in the pleiotropic region. Orange line represents the chromosome threshold and the most significant SNP is highlighted in red. (PDF 9 kb) [file 12864_2018_4598_MOESM7_ESM.pdf]

**MYH1F**


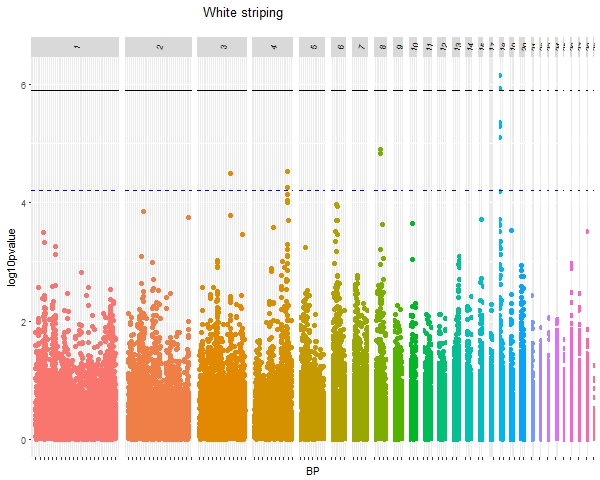

Supplement: Supplementary file 9 — Figure S8. Manhattan plot showing the association of SNPs with MYH1F expression. Black line represents the 5% genome-wide threshold and blue line the 5% GGA18-wide threshold. (DOCX 560 kb) [file 12864_2018_4598_MOESM9_ESM.docx]

**CAV3**


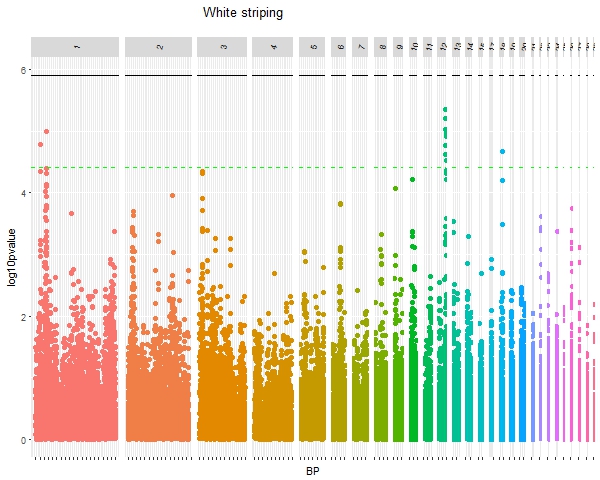

Supplement: Supplementary file 10 — Figure S9. Manhattan plot showing the association of SNPs with CAV3 expression. Black line represents the 5% genome-wide threshold and green line the 5% GGA12-wide threshold. (DOCX 585 kb) [file 12864_2018_4598_MOESM10_ESM.docx]
