# Supplementary material for: Mapping QTL for white striping in relation to breast muscle yield and meat quality traits in broiler chickens
Source: BMC Genomics. 2018 Mar 20;19:202. doi: 10.1186/s12864-018-4598-9 (PMC5859760; doi:10.1186/s12864-018-4598-9)
Supplement: Supplementary file 8 — Table S1. Significant SNPs for the level of expression of the 16 candidate genes. (DOCX 36 kb) [file 12864_2018_4598_MOESM8_ESM.docx]

**Additional file 8: Table S1** Significant SNPs for the level of expression of the 16 candidate genes.

| **Phenotype*** | **eQTL** | **GGA** | **SNP ID** | **Position** (pb)** | ***P*-value***** | ***Corresponding QTL*** |
| --- | --- | --- | --- | --- | --- | --- |
| *MYH15* | eQTL1 | 1 | Gga_rs15350490 | 93828600 | 2.95 x 10^-6^ |  |
| *MYH15* | eQTL1 | 1 | GGaluGA033104 | 93885278 | 2.95 x 10^-6^ |  |
| *DYSF* | eQTL2 | 2 | Gga_rs14138708 | 11017062 | 9.66 x 10^-6^ |  |
| *PNPLA7* | eQTL3 | 2 | Gga_rs14139566 | 12547472 | 1.29 x 10^-6^ |  |
| *DYSF* | eQTL3 | 2 | Gga_rs14139566 | 12547472 | 5.21 x 10^-6^ |  |
| *SGCB* | eQTL4 | 2 | Gga_rs14142253 | 14889365 | **5.52** **x 10^-7^** |  |
| *SGCB* | eQTL5 | 4 | Gga_rs15633060 | 82783597 | 2.02 x 10^-6^ |  |
| *SGCB* | eQTL5 | 4 | Gga_rs14498069 | 82805850 | 1.33 x 10^-6^ |  |
| *FN1* | eQTL6 | 4 | GGaluGA271193 | 90877143 | 1.13 x 10^-5^ | QTL4 |
| *FN1* | eQTL6 | 4 | GGaluGA271252 | 91016447 | 1.38 x 10^-5^ | QTL4 |
| *FN1* | eQTL6 | 4 | Gga_rs13776465 | 91056759 | 6.82 x 10^-6^ | QTL4 |
| *FN1* | eQTL6 | 4 | Gga_rs16454745 | 91098157 | **1.27 x 10^-7^** | QTL4 |
| *MYH13* | eQTL6 | 4 | Gga_rs16454745 | 91098157 | 1.21 x 10^-5^ | QTL4 |
| *FN1* | eQTL6 | 4 | Gga_rs14506759 | 91113465 | **4.33 x 10^-7^** | QTL4 |
| *COL6A3* | eQTL6 | 4 | Gga_rs14506759 | 91113465 | 1.29 x 10^-5^ | QTL4 |
| *FN1* | eQTL6 | 4 | Gga_rs15648179 | 91195923 | **1.21** **x 10^-6^** | QTL4 |
| *COL6A3* | eQTL6 | 4 | Gga_rs15648179 | 91195923 | 1.62 x 10^-5^ | QTL4 |
| *MYH13* | eQTL6 | 4 | Gga_rs15648179 | 91195923 | 1.04 x 10^-5^ | QTL4 |
| *CAPN3* | eQTL6 | 4 | Gga_rs15648179 | 91195923 | 2.85 x 10^-6^ | QTL4 |
| *SGCB* | eQTL7 | 5 | Gga_rs14514700 | 10813149 | 1.45 x 10^-5^ | QTL5 |
| *MYH15* | eQTL8 | 6 | Gga_rs14569880 | 7846186 | **3.59 x 10^-7^** |  |
| *MYH1B* | eQTL9 | 6 | GGaluGA300550 | 18876300 | 2.24 x 10^-5^ |  |
| *MYH1B* | eQTL9 | 6 | GGaluGA300856 | 19644770 | 2.16 x 10^-5^ |  |
| *MYH1B* | eQTL9 | 6 | Gga_rs16004588 | 19885672 | **1.99 x 10^-7^** |  |
| *MYH1B* | eQTL9 | 6 | GGaluGA300927 | 19916374 | 2.53 x 10^-5^ |  |
| *MYH1B* | eQTL9 | 6 | Gga_rs14747990 | 19919971 | 1.20 x 10^-5^ |  |
| *MYH1B* | eQTL9 | 6 | Gga_rs16551619 | 20173119 | 4.72 x 10^-6^ |  |
| *MYH1B* | eQTL9 | 6 | Gga_rs14581509 | 20191350 | **1.12** **x 10^-6^** |  |
| *MYH1B* | eQTL9 | 6 | GGaluGA301022 | 20209447 | 3.47 x 10^-6^ |  |
| *MYH1B* | eQTL9 | 6 | Gga_rs16551732 | 20300076 | 7.30 x 10^-6^ |  |
| *MYH1B* | eQTL9 | 6 | Gga_rs14581613 | 20340618 | 5.41 x 10^-6^ |  |
| *DYSF* | eQTL9 | 6 | Gga_rs14581613 | 20340618 | 2.26 x 10^-5^ |  |
| *PDGFRα* | eQTL9 | 6 | Gga_rs14581613 | 20340618 | 2.58 x 10^-5^ |  |
| *MYH1B* | eQTL9 | 6 | GGaluGA301058 | 20348510 | **6.38 x 10^-7^** |  |
| *MYH1B* | eQTL9 | 6 | Gga_rs14581782 | 20466842 | 1.24 x 10^-6^ |  |
| *MYH1B* | eQTL9 | 6 | Gga_rs15795080 | 20497904 | 1.24 x 10^-6^ |  |
| *MYH1B* | eQTL9 | 6 | Gga_rs16552220 | 20899730 | 7.18 x 10^-6^ |  |
| *MYH1B* | eQTL9 | 6 | Gga_rs14582418 | 21428502 | **3.45 x 10^-7^** |  |
| *MYH1B* | eQTL9 | 6 | Gga_rs16552487 | 21451023 | **1.78 x 10^-7^** |  |
| *MYH1B* | eQTL9 | 6 | Gga_rs14582456 | 21523608 | **2.90 x 10^-7^** |  |
| *MYH1B* | eQTL9 | 6 | GGaluGA301386 | 21789185 | 5.41 x 10^-6^ |  |
| *MYH1B* | eQTL9 | 6 | Gga_rs15796546 | 22181753 | 3.21 x 10^-5^ |  |
| *MYH1F* | eQTL10 | 8 | GGaluGA327044 | 14981718 | 1.24 x 10^-5^ |  |
| *MYH1F* | eQTL10 | 8 | GGaluGA327060 | 15017199 | 1.46 x 10^-5^ |  |
| *FN1* | eQTL11 | 9 | Gga_rs14670735 | 3247110 | 2.86 x 10^-5^ |  |
| *MYHCD* | eQTL12 | 11 | Gga_rs15624232 | 17522949 | 1.72 x 10^-5^ |  |
| *CAV3* | eQTL13 | 12 | GGaluGA089543 | 18654409 | 2.41 x 10^-5^ |  |
| *CAV3* | eQTL13 | 12 | Gga_rs14986262 | 18689560 | 1.67 x 10^-5^ |  |
| *CAV3* | eQTL13 | 12 | Gga_rs14048799 | 18767047 | 6.13 x 10^-6^ |  |
| *CAV3* | eQTL13 | 12 | GGaluGA089616 | 18780013 | 6.13 x 10^-6^ |  |
| *CAV3* | eQTL13 | 12 | Gga_rs14048831 | 18798000 | 6.13 x 10^-6^ |  |
| *CAV3* | eQTL13 | 12 | Gga_rs14048897 | 18828242 | 6.13 x 10^-6^ |  |
| *CAV3* | eQTL13 | 12 | Gga_rs14048907 | 18840435 | 6.13 x 10^-6^ |  |
| *CAV3* | eQTL13 | 12 | Gga_rs15673122 | 18850967 | 6.13 x 10^-6^ |  |
| *CAV3* | eQTL13 | 12 | Gga_rs15673151 | 18869930 | 4.42 x 10^-6^ |  |
| *CAV3* | eQTL13 | 12 | Gga_rs14048959 | 18883392 | 4.42 x 10^-6^ |  |
| *CAV3* | eQTL13 | 12 | GGaluGA089676 | 18904308 | 4.42 x 10^-6^ |  |
| *CAV3* | eQTL13 | 12 | Gga_rs14048983 | 18910817 | 4.42 x 10^-6^ |  |
| *CAV3* | eQTL13 | 12 | Gga_rs13716246 | 18942750 | 4.42 x 10^-6^ |  |
| *CAV3* | eQTL13 | 12 | Gga_rs15673825 | 19090345 | 9.50 x 10^-6^ |  |
| *CAV3* | eQTL13 | 12 | Gga_rs13716306 | 19099718 | 9.50 x 10^-6^ |  |
| *CAV3* | eQTL13 | 12 | Gga_rs14049158 | 19109704 | 9.50 x 10^-6^ |  |
| *CAV3* | eQTL13 | 12 | Gga_rs13716307 | 19119257 | 9.50 x 10^-6^ |  |
| *CAV3* | eQTL13 | 12 | Gga_rs14049196 | 19157040 | 1.09 x 10^-5^ |  |
| *CAV3* | eQTL13 | 12 | Gga_rs14049226 | 19188389 | 1.09 x 10^-5^ |  |
| *CAV3* | eQTL13 | 12 | Gga_rs14049233 | 19202371 | 2.34 x 10^-5^ |  |
| *CAV3* | eQTL13 | 12 | GGaluGA089825 | 19211057 | 2.34 x 10^-5^ |  |
| *CAV3* | eQTL13 | 12 | Gga_rs15675016 | 19468890 | 1.19 x 10^-5^ |  |
| *CAV3* | eQTL13 | 12 | GGaluGA090066 | 19512769 | 1.19 x 10^-5^ |  |
| *CAV3* | eQTL13 | 12 | Gga_rs15675344 | 19678293 | 2.96 x 10^-5^ |  |
| *CAV3* | eQTL13 | 12 | Gga_rs15675477 | 19824102 | 2.96 x 10^-5^ |  |
| *CAV3* | eQTL13 | 12 | GGaluGA090146 | 19873243 | 2.96 x 10^-5^ |  |
| *CAV3* | eQTL13 | 12 | GGaluGA090149 | 19885805 | 2.96 x 10^-5^ |  |
| *TUBB4B* | eQTL14 | 13 | Gga_rs14055240 | 3931630 | 2.22 x 10^-5^ |  |
| *TUBB4B* | eQTL14 | 13 | Gga_rs14055227 | 3946268 | 4.02 x 10^-5^ |  |
| *TUBB4B* | eQTL14 | 13 | GGaluGA091901 | 3952155 | 4.02 x 10^-5^ |  |
| *TUBB4B* | eQTL14 | 13 | Gga_rs14055198 | 3978869 | 2.22 x 10^-5^ |  |
| *LRSAM1* | eQTL15 | 17 | Gga_rs15807988 | 1193728 | 1.08 x 10^-5^ | QTL12 |
| *LRSAM1* | eQTL15 | 17 | GGaluGA112420 | 1341951 | 2.34 x 10^-5^ | QTL12 |
| *LRSAM1* | eQTL15 | 17 | Gga_rs14104648 | 1384574 | **1.65 x 10^-7^** | QTL12 |
| *LRSAM1* | eQTL15 | 17 | GGaluGA112483 | 1437473 | 6.41 x 10^-6^ | QTL12 |
| *LRSAM1* | eQTL15 | 17 | GGaluGA112512 | 1496390 | 1.31 x 10^-5^ | QTL12 |
| *LRSAM1* | eQTL15 | 17 | Gga_rs15034779 | 1497553 | 1.31 x 10^-5^ | QTL12 |
| *LRSAM1* | eQTL15 | 17 | Gga_rs14104352 | 1724666 | **1.52 x 10^-7^** | QTL12 |
| *LRSAM1* | eQTL15 | 17 | Gga_rs15806580 | 1740184 | **5.32 x 10^-7^** | QTL12 |
| *LRSAM1* | eQTL15 | 17 | Gga_rs13734851 | 1756449 | **1.09** **x 10^-6^** | QTL12 |
| *LRSAM1* | eQTL15 | 17 | GGaluGA112716 | 1901705 | **9.65 x 10^-10^** | QTL12 |
| *LRSAM1* | eQTL15 | 17 | GGaluGA112724 | 1916155 | **9.91 x 10^-10^** | QTL12 |
| *LRSAM1* | eQTL15 | 17 | Gga_rs15034195 | 1932600 | **9.55 x 10^-10^** | QTL12 |
| *LRSAM1* | eQTL15 | 17 | Gga_rs15034122 | 1967526 | **1.25 x 10^-7^** | QTL12 |
| *LRSAM1* | eQTL15 | 17 | Gga_rs14104015 | 1985536 | 2.36 x 10^-6^ | QTL12 |
| *LRSAM1* | eQTL15 | 17 | Gga_rs15034085 | 2008784 | **9.47 x 10^-8^** | QTL12 |
| *LRSAM1* | eQTL15 | 17 | Gga_rs15034052 | 2036405 | **2.00 x 10^-7^** | QTL12 |
| *LRSAM1* | eQTL15 | 17 | Gga_rs14103950 | 2071102 | 2.17 x 10^-6^ | QTL12 |
| *LRSAM1* | eQTL15 | 17 | GGaluGA112834 | 2105706 | 2.68 x 10^-6^ | QTL12 |
| *LRSAM1* | eQTL15 | 17 | Gga_rs10728503 | 2196528 | 4.98 x 10^-5^ | QTL12 |
| *LRSAM1* | eQTL15 | 17 | Gga_rs14103847 | 2241378 | 2.58 x 10^-5^ | QTL12 |
| *LRSAM1* | eQTL15 | 17 | Gga_rs14103837 | 2245302 | 1.42 x 10^-5^ | QTL12 |
| *LRSAM1* | eQTL15 | 17 | GGaluGA112954 | 2283348 | **2.15 x 10^-7^** | QTL12 |
| *LRSAM1* | eQTL15 | 17 | GGaluGA112991 | 2317679 | 2.88 x 10^-5^ | QTL12 |
| *LRSAM1* | eQTL15 | 17 | Gga_rs15804842 | 2376482 | 1.33 x 10^-5^ | QTL12 |
| *LRSAM1* | eQTL15 | 17 | GGaluGA113079 | 2433579 | 1.99 x 10^-5^ | QTL12 |
| *LRSAM1* | eQTL15 | 17 | GGaluGA113081 | 2442434 | 1.30 x 10^-5^ | QTL12 |
| *LRSAM1* | eQTL15 | 17 | Gga_rs16753087 | 2656173 | 8.78 x 10^-6^ | QTL12 |
| *LRSAM1* | eQTL15 | 17 | GGaluGA113309 | 2766427 | 4.14 x 10^-5^ | QTL12 |
| *LRSAM1* | eQTL15 | 17 | GGaluGA113338 | 2833719 | 5.41 x 10^-5^ | QTL12 |
| *MYH1F* | eQTL16 | 18 | Gga_rs15809155 | 297096 | 5.03 x 10^-6^ |  |
| *MYH1F* | eQTL16 | 18 | Gga_rs14105451 | 306139 | 5.03 x 10^-6^ |  |
| *MYH1F* | eQTL16 | 18 | Gga_rs14105472 | 343079 | 4.40 x 10^-6^ |  |
| *MYH1F* | eQTL16 | 18 | GGaluGA117789 | 343205 | 5.03 x 10^-6^ |  |
| *MYH1F* | eQTL16 | 18 | Gga_rs15809447 | 469140 | 5.03 x 10^-6^ |  |
| *MYH1F* | eQTL16 | 18 | Gga_rs15809547 | 517541 | 5.03 x 10^-6^ |  |
| *MYH1F* | eQTL16 | 18 | Gga_rs14105637 | 523233 | 5.03 x 10^-6^ |  |
| *MYH1F* | eQTL16 | 18 | Gga_rs15035593 | 544688 | 5.03 x 10^-6^ |  |
| *MYH1F* | eQTL16 | 18 | Gga_rs14105707 | 583202 | **7.08 x 10^-7^** |  |
| *MYH1F* | eQTL16 | 18 | Gga_rs14105726 | 601151 | 4.40 x 10^-6^ |  |
| *MYH1F* | eQTL16 | 18 | Gga_rs14105739 | 610899 | **1.17** **x 10^-6^** |  |
| *MYH1F* | eQTL16 | 18 | Gga_rs15809816 | 619881 | 4.53 x 10^-6^ |  |
| *MYH1F* | eQTL16 | 18 | Gga_rs15810060 | 699832 | 5.03 x 10^-6^ |  |
| *MYH1F* | eQTL16 | 18 | Gga_rs13506093 | 828426 | 7.91 x 10^-6^ |  |
| *CAV3* | eQTL17 | 18 | Gga_rs15826142 | 6504359 | 2.12 x 10^-5^ |  |
| *CAV3* | eQTL17 | 18 | Gga_rs14112328 | 6532115 | 6.24 x 10^-5^ |  |
| *LRSAM1* | eQTL18 | 22 | GGaluGA186934 | 2899623 | 1.67 x 10^-5^ |  |
| *PNPLA7* | eQTL18 | 22 | GGaluGA186934 | 2899623 | 2.78 x 10^-5^ |  |
| *TUBB4B* | eQTL18 | 22 | GGaluGA186934 | 2899623 | 1.12 x 10^-5^ |  |
| *LRSAM1* | eQTL18 | 22 | GGaluGA186952 | 2939179 | 2.36 x 10^-5^ |  |
| *PNPLA7* | eQTL18 | 22 | GGaluGA186952 | 2939179 | 1.06 x 10^-4^ |  |
| *TUBB4B* | eQTL18 | 22 | GGaluGA186952 | 2939179 | 1.48 x 10^-6^ |  |
| *TUBB4B* | eQTL18 | 22 | GGaluGA186964 | 2968318 | 1.94 x 10^-5^ |  |
| *MYH1E* | eQTL19 | 23 | Gga_rs14289870 | 3040351 | 1.24 x 10^-5^ |  |
| *MYHCD* | eQTL20 | 24 | Gga_rs14293376 | 1575682 | 1.51 x 10^-5^ |  |
| *MYH1B* | eQTL21 | 27 | Gga_rs16207951 | 3938987 | 8.56 x 10^-5^ |  |

Co-localization with QTL associated with WS and meat quality traits is indicated in the last column.

* *MYH15* myosin heavy chain 15, *DYSF* dysferlin, *PNPLA7* patatin-like phospholipase domain containing 7, *SGCB* sarcoglycan beta, *FN1* fibronectin 1, *MYH13* myosin heavy chain 13, *COL6A3* collagen type 6 alpha 3, *CAPN3* calpain 3, *MYH1B* embryonic myosin heavy chain 3, *PDGFRα* platelet derived growth factor receptor alpha, *MYH1F* neonatal myosin heavy chain, *MYHCD* myosin heavy chain CD, *CAV3* caveolin 3, *TUBB4B* tubulin beta 4B class IVb, *LRSAM1* leucine rich repeat and sterile alpha motif containing 1, *MYH1E* adult myosin heavy chain

** Positions are indicated on galgal5 assembly

*** Genome-wide significant *P*-values are indicated in bold
