# Supplementary material for: Mapping QTL for white striping in relation to breast muscle yield and meat quality traits in broiler chickens
Source: BMC Genomics. 2018 Mar 20;19:202. doi: 10.1186/s12864-018-4598-9 (PMC5859760; doi:10.1186/s12864-018-4598-9)
Supplement: Supplementary file 11 — Table S2. Primer sequences of the 16 candidate genes and the housekeeping gene (PDE3B). (DOCX 14 kb) [file 12864_2018_4598_MOESM11_ESM.docx]

**Additional file 11: Table S2** Primer sequences of the 16 candidate genes and the housekeeping gene (PDE3B)

| **Gene** | **Gene ID** | **Forward primer** | **Reverse primer** |
| --- | --- | --- | --- |
| *MYH15* | 395534 | TTCAAGCAAACCCAGCCCTA | GATTTTTCAGCAACCGGGTG |
| *PDGFRα* | 395509 | GAGTCACAAAAGCCGTGGACAT | TGTTTTCTCACAGGACCGCTC |
| *SGCB* | 422760 | TGTAGAGCGCAGGAACGTCAAT | AATCACCGCCCAGATAACGAG |
| *COL6A3* | 396548 | AGCCCAAAGTGACCTACACG | GCAGATGTCCATGACATTTTCAG |
| *FN1* | 396133 | CACAAACACCAACGTCAACT | GTTTGGATGGTAATCGTGGCAG |
| *LRSAM1* | 417265 | AGGTCCTCAATGTGAAAGGCA | TTGCAGAGGAACTGCTGAATG |
| *PNPLA7* | 427774 | TTTCACCATCAAGGCCAATCG | GTCCAGCCTCAACTTCCATCCA |
| *TUBB4B* | 417255 | CAGACCGGATTATGAACACCTT | CACCGTATGTTGGCGTAGTT |
| *MYH13* | 768487 | GGAATGACAACTCCTCACGCT | GCTAGCTGGAAAGTCACTCTGG |
| *MYH1B* | 374069 | AGGAGCTGTCCAATGTCAACCTC | GCAGAAGAAAGCAACAGAGGGTTC |
| *MYH1E* | 427788 | CCAAATTCCGCAAGATCCAAC | CTTATGCCACTTTGTTGTCACGAC |
| *MYH1F* | 768566 | AGCTGTCCAATGTCAACCTTTCC | TGCCTCAGGTCACACTTTAGC |
| *MYOCD* | 427790 | GTCTGAGCACTCCTTGCTGATT | CGTTGTTCATGGAATGCGG |
| *DYSF* | 425353 | GGTCGGGATGAGCCAAACAT | GTTCGGGAAGGCGTAGATGA |
| *CAPN3* | 423233 | CAAACCAGTGCTCATTCCCT | GCCTGACCCACACTGATTTT |
| *CAV3* | 378796 | GAAAGGCAGCTACACCACCT | GTAGATGCGGCTGACACACT |
| *PDE3B* | 423064 | CACCATCTCAGCGAAAATCACA | TCTCCTAGCTGCCGCTCATCTT |
